# TickPath Layerplex: adaptation of a real-time PCR methodology for the simultaneous detection and molecular surveillance of tick-borne pathogens

**DOI:** 10.1038/s41598-019-43424-y

**Published:** 2019-05-06

**Authors:** Joseph J. Modarelli, Pamela J. Ferro, Adalberto A. Pérez de León, Maria D. Esteve-Gasent

**Affiliations:** 1 Department of Veterinary Pathobiology, College of Veterinary Medicine and Biomedical Sciences, Texas A&M University, College Station, TX 77843 USA; 2 Texas A&M Veterinary Medical Diagnostic Laboratory, Texas A&M University, College Station, TX 77843 USA; 3 USDA–ARS Knipling–Bushland U.S. Livestock Insects Research Laboratory and Veterinary Pest Genomics Center, Kerrville, TX 78028 USA

**Keywords:** Microbiology techniques, Infectious-disease diagnostics

## Abstract

Tick-borne diseases (TBD) are common across the United States and can result in critical and chronic diseases in a variety of veterinary patients. Moreover, borreliosis, anaplasmosis, rickettsiosis, ehrlichiosis, and babesiosis are zoonotic and have been cited as the most common TBDs. Molecular diagnostic methodologies utilized for screening domestic dogs for these causative agents include real-time PCR (qPCR) assays in both singleplex and multiplex formats. However, current limitations of qPCR instruments restrict the number of fluorogenic labels that can be differentiated by the instrument for a given reaction. This study describes the development of the TickPath Layerplex, a diagnostic assay based on qPCR methodology that was adapted for the simultaneous detection and characterization of 11 pathogens responsible for causing 5 common TBDs in domestic dogs. The analytical and diagnostic performance of the layerplex assay was evaluated and shown to be compatible with common instruments utilized in molecular diagnostic laboratories. Test results revealed no inhibition or reduction in sensitivity during validation of the layerplex assay, and the limit of detection was determined to be near 16 genome copy equivalents per microliter. Overall, the high sensitivity, specificity, and screening capability of the assay demonstrate its utility for broadly screening dogs for common TBDs.

## Introduction

Tick-borne pathogens, including *Borrelia hermsii* (Bh), *B*. *turicatae* (Bt), *B*. *parkeri* (Bp), *B*. *burgdorferi* (Bb), *Ehrlichia canis* (Ec), *E*. *chaffeensis* (Ech), *E*. *ewingii* (Ee), *Anaplasma phagocytophilum* (Ap), and *Rickettsia rickettsii* (Rr) infect dogs and humans, are zoonotic, and are considered a growing public health problem^1–4^. Tick-borne diseases (TBD) affecting domestic dogs are prevalent throughout the United States and are regarded as an emerging infectious threat^1^. The risk of exposure to some of these pathogens, such as Ec in humans or Bp in both dogs and humans, remains to be fully recognized because documentation of infection in patients is limited^3,5^. Additionally, species of tick-borne protozoa in the genus *Babesia* (Bab) have a worldwide distribution and infection can result in various levels of clinical severity^6^. Of most concern to U.S. dogs are *B*. *gibsoni* and *B*. *canis vogeli*, though *B*. *conradae* has also recently been implicated in canine babesiosis^7,8^.

Various tick species serve as vectors of several bacterial, viral, and protozoal pathogens causing disease in humans, domestic animals, and wildlife. Proven primary vectors for the assortment of pathogens discussed here include various species in the Ixodidae (hard ticks) and Argasidae (soft ticks) tick families^2^. Wildlife are generally considered maintenance hosts, with dogs and humans acting as incidental hosts for the tick vectors^2,3^. Dogs have been considered sentinels for the risk of human exposure to infected tick vectors and may signify geographical regions of increased zoonotic risk because of their close environmental association with humans^9,10^.

Universally accepted tests remain to be developed for all TBDs affecting dogs, yet diagnosis primarily relies on several serological assays (ELISA, immunofluorescence, and immunoblot) and blood smear examination^10–12^. Although clinical signs can be observed within days of infection, detectable antibody production can take up to 28 days^13^. In some cases, dogs do not generate an antibody response of a large enough magnitude to be detectable by the currently available serological assays, even when clinical signs are present^14^. In dogs, clinical signs are similar among several tick-borne diseases, thus deducing a diagnosis would require multiple serological panels and result in a delay in effective treatment. High antibody titers may persist for months to years following clinical resolution of some tick-borne pathogens, limiting the value of monitoring titers during treatment and confounding the detection of repeat or concurrent infections^14,15^. While serologic assays for diagnosing TBDs in veterinary patients are commonly used and provide valuable information, molecular diagnostic tools (e.g. PCR), in contrast, are often used for detecting acute infections and monitoring responses to treatment due to detecting the presence of the pathogen’s DNA. However, some TBD pathogens only circulate in blood in limited quantities during the acute phase of infection, as is the case for Bb and Rr^16,17^. PCR may be valuable in revealing an infection prior to seroconversion but may lose its effectiveness as the infection persists. Therefore, the limitations of molecular and serological diagnostic tools should be recognized and may be most effective in determining a differential diagnosis when utilized in parallel^14^.

Newer molecular assays permit the addition of fluorogenic probes (e.g. quantitative real-time PCR, or qPCR) that allows for a technique known as multiplexing, which has proven to be a useful tool in screening an animal sample for more than one TBD pathogen at a time^18,19^. Since many TBDs can present with similar clinical signs or as co-infections, panels detecting multiple pathogens would be ideal for comprehensive pathogen identification^20^. However, current qPCR platforms limit the number of probes that can be detected by a given instrument, which subsequently limits the number of pathogens that can be screened in a single test. Most of the widely-utilized qPCR platforms are limited to multiplexing 4–5 fluorophores in a single reaction without interfering with pathogen detection. Current approaches to molecular screening for more than four to five targets at a time require the testing to be conducted in more than one multiplex panel. Consequently, comprehensive molecular testing of a single sample increases associated costs by requiring more reagents and possibly, depending on the number of instruments available, additional time to reach diagnoses.

For these reasons, the development of an affordable molecular screening test for a broad array of pathogens is desirable. Many of the molecular assays currently employed for detecting these TBDs in dogs include various conventional PCR (gel based), singleplex qPCR (single target), or multiplex qPCR assays (multiple targets dependent on instrument capabilities)^18,19,21–25^. However, a typically unutilized, yet perhaps familiar, technique that allows numerous pathogens to be detected in a single qPCR reaction is the process of assigning more than one target pathogen to a single fluorogenic label, as briefly demonstrated in a previous study^26^. While colloquially referred to as typical qPCR multiplexing, this study proposes a terminology distinction to differentiate this qPCR technique, herein referred to as layerplexing.

A differentiating aspect of layerplexing involves utilizing any probe to label more than one target assay in a given reaction. In contrast, general qPCR multiplexing uses the same probe but for only labeling a single target assay in a reaction. Layerplexing would allow for a large number of unique target assays to be grouped under each probe label, up to the qPCR instruments fluorogenic limit (e.g. 11 targets under 4 probe labels). Once a sample has been screened using the layerplex technique (initial layer), subsequent testing using singleplex or multiplexing versions of each target assay would then be necessary to identify the specific pathogen(s) responsible for infection depending on which initial probe indicated amplification. While additional testing is required after the initial layer to characterize positive species, a negative layerplex result would indicate that the sample was negative for all pathogens screened. This increased screening capability enhances not only animal diagnostics, but also surveillance studies. Further, the layerplex technique achieves high-throughput testing for all targets by utilizing a single DNA extraction and one qPCR reaction, effectively circumventing current qPCR platform’s unique-probe limitations while maintaining compatibility with commonly utilized qPCR instruments.

This study aimed to design the molecular diagnostic tool we termed the TickPath Layerplex that uses the qPCR layerplexing technique to screen for the most common TBDs affecting domestic dogs by simultaneously targeting a broad spectrum of pathogens known to cause TBDs. To this end, the pathogenic species that are of most concern to domesticated dogs within five TBD groups (i.e. borreliosis, anaplasmosis, rickettsiosis, ehrlichiosis, and babesiosis) were determined based on past prevalence studies and subsequently targeted for qPCR assay development^10,27–31^. Overall, this study details the design and validation of 10 singleplex assays for each of the targeted pathogens, and the subsequent validation of integrating 11 assays into a single layerplex qPCR assay.

## Results

### Evaluation of layerplex oligonucleotides

The TickPath Layerplex assay was developed for the detection of Bh, Bt, Bp, Bb, Ec, Ech, Ee, Ap, Rr, and pan-Bab species in domesticated dog diagnostic samples. Each pathogen specific set of oligonucleotides was optimized independently, and then layered together based on species similarity into a single-reaction assay. A canine specific endogenous internal positive control (EIPC-K9) was utilized in the consolidated layerplex assay^32^. The sequences of all oligonucleotides, their reaction concentrations, and their respective target genes are detailed in Table 1.

**Table 1:** Tick-borne pathogen assays organized by layers and endogenous internal positive control (EIPC-K9) polymerase chain reaction primers and probes sequences, amplicon sizes, and oligonucleotide concentrations.

Layer/Oligonucleotide	Sequence (5′-3′) and reporter dye*	Amplicon size (bp)	Final concentration (nM)	Target region	Reference
Borrelial					
Bb.flaB.161F	AAGAGGGTGTTCAACAGGAAGG	75	450	*flaB*	This study
Bb.flaB.213R	GAGAATTAACTCCGCCTTGAGAA		450		
Bb.flaB.186P	FAM-TCAACAGCCAGCACCTGCTACAGCA-BHQ1		125		
Bh.flaB.531F-1	GGGCGCAAATCAGGATGAG	119, 117	450	*flaB*	This study &^33^
Bh.flaB.529F-2	GTGGGAGCAAATCAGGATGAG		450		
Bh.flaB.647R	TCCTCTTGCTGTCCTATCTCTTGC		450		
Bh.flaB.615P	FAM-AGCCTGAGCRCCTTCACCTGCAAAAAGA-BHQ1		125		
Bt.bipA.728F-1	AGACCGGTACACAGGATTCTAAAGC	139, 142	450	*bipA*	This study
Bt.bipA.731F-2	CCGGCACACAGGATTCTAAAAC		450		
Bt.bipA.869R	GTTCCTGCTCCCTGAATAACATTATC		450		
Bt.bipA.818P	FAM-AGTTTTGGGAAGTGTTGTTGGTGGCGT-BHQ1		125		
Bp.flaB.406F	TTGTCCAATAAGTCAGCTGCTCAG	117	450	*flaB*	This study
Bp.flaB.522R	TCTTAATGTCCATGAAGCTTGTGC		450		
Bp.flaB.443P	FAM-CTGAAGAGCTTGGAATGCAACCTGCA-BHQ1		125		
Rickettsial					
Rr.hyp.724702F	AGAGTAAATCAACGGAAGAGCAAAAC	159	450	*rrhyp*	This study
Rr.hyp.724860R	CCCCTCCACTACCTGCATCAT		450		
Rr.hyp.724788P	CFO560-TCCTCTCCAATCAGCGATTCAGGCA-BHQ1		125		
Ap.msp2.420F	GACTTTCCTAGCATGGAGTTGGTT	95	450	*msp2*	This study
Ap.msp2.514R	GCGTGCCCTTTTGTAATACCTATAA		450		
Ap.msp2.452P	CFO560-CATTTCACCTTACACATGCGCCGGA-BHQ1		125		
Ech.16S.64F	GAACGGACAATTGCTTATAACCTTTT	111	450	*16S rRNA*	This study
Ech-Ee.16S.174R	CCATCATTTCTAATGGCTATTCCATACT		450		
Ee.16S.40F	CGAACGAACAATTCCTAAATAGTCTCT	114	450		
Ec.16S.61F	GCCTCTGGCTATAGGAAATTGTTAGT	113	450		
Ec.16S.148R	CTCGGGGATTATACAGTATTACCCAC		450		
Ehrl-spp.16S.83P	CFO560-AGATTCCTACGCATTACTCACCCGTCTGC-BHQ1		125		
Babesial					
Babsp.18S.65F-1	CGCATTTAGCGATGGACCA	94, 93	450	*18S rRNA*	This study
Babsp.18S.67F-2	GCTTTTAGCGATGGACCATTCA		450		
Babsp.18S.289R	CCTAATTCCCCGTTACCCGTT		450		
Babsp.18S.228P	Q670-CATCAGCTTGACGGTAGGGTATTGGCC-BHQ2		125		
EIPC-K9					
EIPC.K9mt.12942F	GGATTCTACTCCAAAGACCTGATCA	96	31.25	*MT*-*ND5*	^32^
EIPC.K9mt.13018R	GGTTAGGGATGTGGCAACGA		31.25		
EIPC.K9mt.12980P	TAM-CACGTCGAATACCAACGCCTGAGCC-BHQ1		31.25		

*FAM = 6-carboxyfluorescein; CFO560 = CAL Fluor Orange 560; Q670 = Quasar670; TAM = N,N,N′,N′-tetramethyl-6-carboxyrhodamine; BHQ1&2 = black hole quencher 1&2.

### qPCR linear dynamic range, efficiency, and analytical sensitivity

**Figure 1:**
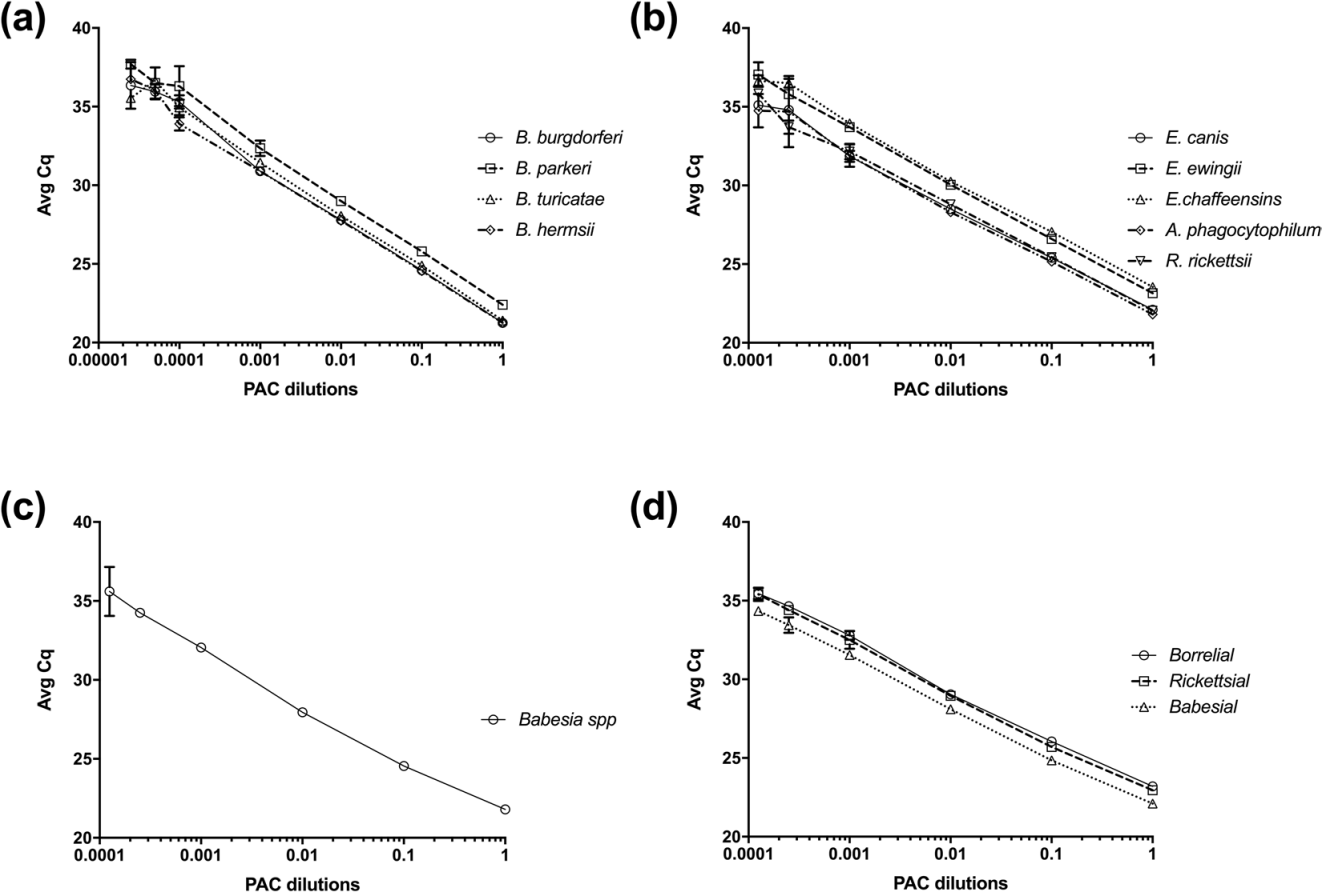
Linear regression plots representing analytical sensitivity of singleplex and layerplex real-time polymerase chain reaction (qPCR) for (**a**) *Borrelia* species (*B*. *burgdorferi*, *B*. *parkeri*, *B*. *turicatae*, *B*. *hermsii*), (**b**) Rickettsiales (*Ehrlichia canis*, *E*. *ewingii*, *E*. *chaffeensis*, *Anaplasma phagocytophilum*, *Rickettsia rickettsii*), (**c**) *Babesia* species, and (**d**) combined pathogen layers (borrelial, rickettsial, and babesial) detection. Plasmid positive amplification control (PAC) was serial diluted and evaluated by qPCR for each singleplex and combined layerplex assays. Each dilution was assessed in duplicate.

The linear dynamic range and efficiency of the layerplex qPCR assay, using the listed primers and probes (Table 1), was established using serial dilutions of the plasmid positive amplification control (PAC) as demonstrated previously^33^. For each pathogen in the layerplex assay analytical sensitivity was evaluated against the respective single pathogen assays. This comparison approach was performed to evaluate the multiple oligonucleotides’ combinatory effects on performance. Analytical sensitivities of the layerplex and singleplex assays were equivalent for the detection of all pathogens as depicted in the linear regression plots (Fig. 1a–d). Results were obtained in duplicate experiments to validate the results provided in Fig. 1. The singleplex and layerplex assays exhibited 91–105% efficiency (R^2^ > 0.99) for the serial dilutions of each target (Table 2). Copy number calculations for limit of detection (LOD) of the qPCR were found to be roughly 16 genome copy equivalents per microliter (GCE/μL) of each target using linear regression analysis and serial dilutions of the plasmid PAC; these were evaluated independently by singleplex and combined by layerplex (Supplementary Tables S1–4).

**Table 2:** Amplification efficiencies for the singleplex and layerplex assays; all R^2^ values > 0.99.

Plex	Assays	Efficiency (%)
Layerplex	Borrelial layer	105%
	Rickettsial layer	104%
	Babesial layer	105%
Singleplex	Bh	99%
	Bt	101%
	Bp	99%
	Bb	97%
	Ap	95%
	Rr	97%
	Ec	97%
	Ech	94%
	Ee	91%
	Bab	91%

### Analytical and *in silico* specificity analysis

Analytical specificity was evaluated by indicating failure to amplify representative species reference controls (i.e. genomic DNA and gBlocks) in duplicate testing from each species other than the intended targets. All homologous and heterologous reference controls used for analytical specificity analysis are detailed in Table 3. Further, through *in silico* analysis of respective gene sequences available through GenBank^®^, each assay’s oligonucleotides were manually evaluated against homologous and heterologous species alignments to verify oligonucleotide mismatch analysis against all available strains and isolates gene sequences (Supplementary Fig. S1). There was no off-target amplification observed through analytical or *in silico* analysis from any borrelial or rickettsial assay in both layerplex and singleplex formats, thus revealing the high specificity of the assays. The babesial layer also demonstrated high specify to intended targets as a pan-*Babesia* assay for canine, equine, bovine and cervine specific species (Table 3).

**Table 3:** Analytical specificities of each layer in the real-time PCR assay.

Panel	No. of samples tested	Layer Result		
		Borrelial	Rickettsial	Babesial
Borrelial target species^a^	5	Positive	Negative	Negative
*Borrelia* near neighbors^b^	7	Negative	Negative	Negative
Rickettsial target species^c^	5	Negative	Positive	Negative
Rickettsiales near neighbors^d^	6	Negative	Negative	Negative
Babesial target species^e^	6	Negative	Negative	Positive
*Babesia* near neighbors^f^	8	Negative	Negative	Negative

Genomic DNA utilized from each pathogen was determined to be at a concentration of approximately 2,000 genome copy equivalents per microliter (GCE/μL).

^a^*Borrelia hermsii* GGI, *B*. *hermsii* GGII, *B*. *turicatae*, *B*. *parkeri*, and *B*. *burgdorferi*.

^b^*B*. *miyamotoi*, *B*. *coriaceae*, *B*. *anserina*, *B*. *crocidurae*, *B*. *recurrentis*, *B*. *garinii*, and *B*. *afzelii*.

^c^*Ehrlichia canis*, *E*. *chaffeensis*, *E*. *ewingii*, *Anaplasma phagocytophilum*, *and Rickettsia rickettsii*.

^d^*E*. *ruminantium*, *E*. *muris*, *A*. *marginale*, *A*. *centrale*, *A*. *ovis*, and *R*. *typhi*.

^e^*B*. *canis*, *B*. *gibsoni*, *B*. *caballi*, *B*. *odocoilei*, *B*. *divergens*, and *B*. *bigemina*.

^f^*Babesia conradae*, *B*. *microti*, *B*. *duncani*, *B*. *bovis*, *Theileria equi*, and *Cytauxzoon felis*.

*Rhipicephalus sanguineus*, *Ixodes scapularis*.

### Inhibitory analysis

**Figure 2:**
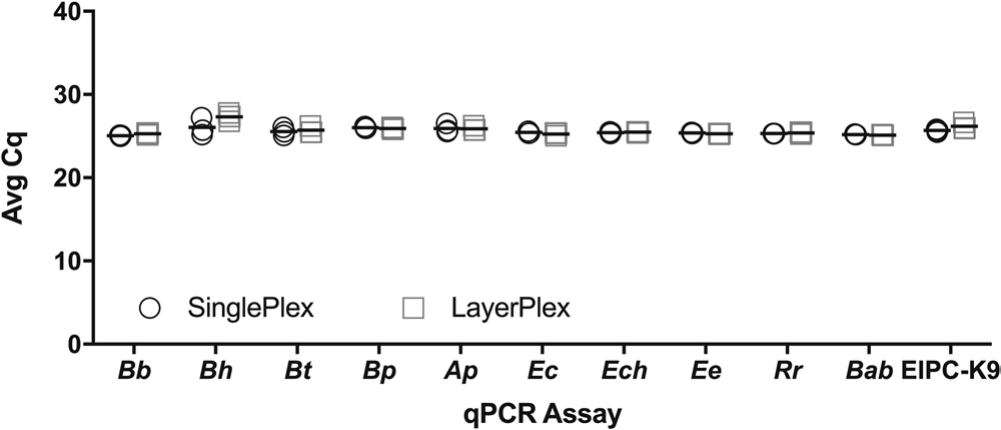
Comparison analysis of singleplex real-time PCR (qPCR) assays quantification cycle (Cq) values against combined layerplex qPCR assay Cq values. Pathogens assayed are as follows: *Borrelia burgdorferi* (Bb), *B*. *hermsii* (Bh), *B*. *turicatae* (Bt), *B*. *parkeri* (Bp), *Anaplasma phagocytophilum* (Ap), *Ehrlichia canis* (Ec), *E*. *chaffeensis* (Ech), *E*. *ewingii* (Ee), *Rickettsia rickettsii* (Rr), *Babesia* species (Bab), and endogenous internal positive control (EIPC-K9). Singleplex qPCR conditions contained only the primers and probes needed for respective testing. Layerplex qPCR conditions contained all primers and probes listed in Table 1. All assays (singleplex and layerplex) were tested in triplicate against the same respective genomic DNA for each species. A paired t-test was conducted to statistically analyze Cq values obtained from both assay conditions.

Through triplicate testing, quantification cycle (Cq) values derived from qPCR analysis were comparable for the detection of all species by singleplex testing and the detection of the same species in layerplex format. Further, there was no statistically significant inhibition (p > 0.05) between either assay conditions by paired t-test statistical analysis of mean Cq values (Fig. 2, Supplementary Tables S11–13). Results also indicated that the EIPC-K9 did not experience significant inhibition in the layerplex format when compared to the singleplex version (Supplementary Table S14).

### Layerplex qPCR performance evaluation using randomly collected animal diagnostic samples

A collection of 1,171 blood samples from domestic dog (*Canis lupus familiaris*), 211 brown dog ticks (*Rhipicephalus sanguineus*), 35 black-legged ticks (*Ixodes scapularis*), and 18 tissues (9 skin and 9 joint) from 9 mice (*Mus musculus*) infected with *Borrelia burgdorferi* B31 A3, were assayed with the TickPath Layerplex to determine diagnostic sensitivity and specificity. Analysis revealed a total of 26, 37, and 5 positives from borrelial, rickettsial, and babesial layers, respectively. Positive samples at the layered level were then subjected to relevant singleplex analysis to determine species identity, with the exception of the 5 babesial layer positives due to the pan-*Babesia* spp. screening nature of the assay in which conventional PCR alone was used for species identification. Pathogen species identified from the diagnostic sample set included *Ehrlichia canis* (19 from dogs, 18 from brown-dog ticks), *Borrelia turicatae* (8 from dogs), *B*. *burgdorferi* (9 joint and 9 skin from mice), and *Babesia gibsoni* (5 from dogs). All positive samples were confirmed through relevant conventional PCR and Sanger sequencing. Diagnostic analysis at a Cq value cutoff of 38 revealed sensitivity and specificity values (including 95% confidence intervals) of 100% (86.8–100%) and 99.8% (99.4–99.9%), 100% (90.5–100%) and 99.1% (98.4–99.5%), and 100% (47.8–100%) and 100% (99.7–100%) for borrelial, rickettsial, and babesial layers, respectively (Table 4). Singleplex analysis of relevant pathogen assays revealed similar results. EIPC-K9 detected 100% of the dog DNA screened in this study at a mean Cq value of 21.2, and all sample values were within the appropriate range^32^. All negative tick samples, and a randomly selected set of 50 negative dog samples, were subjected to the aforementioned conventional PCR assays to confirm absence of relevant pathogen species.

**Table 4:** Diagnostic test evaluation of the layerplex assay from analyzed sample set.

	Borrelial	Rickettsial	Babesial
True positives	26	37	5
False Positives	3	13	0
False Negatives	0	0	0
True Negatives	1396	1375	1420
Sensitivity	100% (86.8–100%)	100% (90.5–100%)	100% (47.8–100%)
Specificity	99.8% (99.4–99.9%)	99.1% (98.4–99.5%)	100% (99.7–100%)

Values are displayed with percentages and 95% confidence intervals.

## Discussion

Early detection and pathogen identification are critical to limit the impact of borreliosis, anaplasmosis, ehrlichiosis, rickettsiosis, and babesiosis on an infected canine patient. The ability to use a single sample and test to screen for all 5 infections simultaneously and rapidly is highly desirable to reach the correct diagnosis. The main benefit of the newly termed TickPath Layerplex assay, described herein, includes the ability to screen for all 10 pathogen specific assays concurrently without a reduction in sensitivity or specificity compared to singleplex analysis. The method described herein also allows for an EIPC to be utilized, despite already targeting numerous pathogens, due to the consolidation of probe labels. Though this study utilized an EIPC, an exogenous internal positive control (XIPC) could be substituted and probe labeled accordingly.

While the detection of a positive layer from qPCR testing would require additional conventional, singleplex and/or multiplex testing to reveal the specific pathogen species identity, a negative result obtained by the same method would not require additional testing and instead indicate the lack of pathogen presence in the screened sample. As treatment is similar for all pathogens screened within the respective layers, diagnosticians and veterinarians may find that additional singleplex testing is not necessary to determine the appropriate patient treatment. Although treatment can be determined based on results at the layer level, it is strongly recommended that positive results be subsequently identified to the species level due to public health concern with reportable pathogens included in the screen (e.g. Rocky Mountain spotted fever, Lyme disease, etc.).

Oligonucleotides designed for qPCR detection of the aforementioned tick-borne diseases have resulted in a unique look into the specificity of oligonucleotides under various mismatch conditions. Though the intention of this study was not to determine the exact number of mismatches needed to support efficient qPCR differentiation between species, data presented here does provide insightful evidence for future qPCR assays aiming to differentiate between genetically similar species. As depicted in Supplemental Fig. S1C, for example, a minimum of 1–2 mismatches in a general primer location, and 1 in the 3′ position of a primers annealing location, was sufficient in restricting primer annealing on a heterologous species, and therefore preventing the extension of DNA polymerase and subsequent formation of an undesired amplicon. Additionally, in the case of *Ehrlichia* species detection, analysis revealed that although the probe sequence used for the detection of all *Ehrlichia* species is identical, differentiation can be achieved at the primer level, due in part, to the presence of significant mismatches. Further, differentiation was achieved even when the probe and reverse primer sequence were identical, as is the case with *Ehrlichia chaffeensis* and *E*. *ewingii*, and *Borrelia turicatae* and *B*. *parkeri* (Supplemental Fig. S1C–F, respectively). Here, species differentiation was achieved by placing the forward primer over detected mismatches, and designing the probe to anneal on the species’ sense strand only. In this case, the mismatches of the forward primer prevent the DNA polymerase from successfully annealing and cleaving the probe that would anneal onto heterologous species, and only provide detection for the appropriate species.

In specific situations, a number of assays described in this study utilized multiple forward primers. The utilization of more than one forward primer was necessary to anneal and subsequently detect all strain variants within the intended species. *Borrelia hermsii*, for example, has been described to contain two unique genomic groups (GG), which differ slightly genetically^34^. Therefore, the assay designed to detect the pathogen required two forward primers to compensate for the additional mismatches within the *flaB* gene of the two GG (Supplemental Fig. S1B). A similar technique was also required for efficient detection *of B*. *turicatae* (Supplemental Fig. S1D). In the case of the pan-*Babesia* assay, two forward primers were utilized in order to detect all canine specific species desired with one assay (Supplemental Fig. S1J). The pan-*Babesia* assay proved efficient in amplifying a number of additional species including the desired canine specific pathogens. Although there are mismatches present in the additional *Babesia* species detected by the assay, the mismatches are mostly situated on the 5′ end of the forward primer and was not found to negatively affect primer binding activity. Consequently, the pan-*Babesia* assay allowed for a more thorough screening of a sample for a more complete *Babesia* species repertoire. Future studies should seek to define limitations of the assay in respect to broad-spectrum *Babesia* species not covered in this study.

The goal of this study was to evaluate the layerplex methodology for targeting 11 targets (i.e. 10 tick-borne pathogens and an EIPC) in terms of performance (i.e. LOD, efficiency, and R^2^), and specificity to a wide range of homologous and heterologous species reference controls. In that respect, performance of the layerplex qPCR was comparable to other singleplex and duplex qPCR assays available for the detection of tick-borne pathogens^17–19,21–25^. Efficiency and R^2^ values for all assays in both singleplex and layerplex formats were within ideal ranges and were shown to be unaffected by the large collection of oligonucleotides present in the assay. In analytical sensitivity testing, the layerplex displayed no loss in sensitivity in any layer when compared to the respective singleplex qPCR assays. Further, no oligonucleotide-induced inhibition was observed when comparing the detection capabilities of the layerplex format to singleplex testing. As the layerplex assay depicted in this study revealed efficient detection of 11 targets labeled with 4 probes in a layerplex format, further studies are warranted to evaluate the potential of detecting more targets than the limited analysis presented here.

Although linear regression analysis revealed that a Cq value of 36 was an adequate positive sample cutoff (Supplementary Tables S1–4), diagnostic sample analysis indicated a number of false negatives at that cutoff value. When the Cq cutoff value was increased to 38, sensitivity evaluations increased to 100%, and eliminated the occurrence of false negatives. Therefore, a Cq of 38 should be considered the cutoff value for diagnostic samples in order to detect all potentially weak positive samples. It is important to note that analyzing samples in terms of a Cq value of 38 increases sensitivity at the expense of specificity. Samples presenting Cq values near the cutoff value should be assessed in conjunction with additional diagnostic modalities (i.e. serological findings, clinical presentation, etc.). Further, due to the lack of identified positive samples for various TBD species (e.g. *Babesia canis*, *Borrelia turicatae*, *Ehrlichia chaffeensis*, etc.) current validation analysis can only estimate the diagnostic specificity and sensitivity of these assays until further studies are conducted. However, the provided analytical and *in silico* analysis for each assay, accompanied with the diagnostic performance of the respective layered assays, should be used as a guideline until additional positive diagnostic samples are tested in subsequent studies. Though no coinfections were observed from the available diagnostic sample data set, the ability of the layerplex assay to support parallel detection through amplification of multiple probe dyes is supported by the linear regression analysis that depicted results for all layers labeled independently with unique probe dyes during concurrent testing.

Access to additional reference controls was limited, resulting in substantial reliance on *in silico* analysis of publicly available nucleotide sequences. It is important to note that additional *Rickettsia rickettsii* reference controls, and controls representing near-neighbors (i.e. *R*. *parkeri*, *R*. *akari*, etc.), were not available for evaluation. Additionally, as the *R*. *rickettsii* assay is targeting a hypothetical protein conserved in *R*. *rickettsii* strains only (hypothetical protein A1G_04230), available GenBank^®^ sequences were also limited for *in silico* analysis. However, *in silico* sequence analysis of the *R*. *rickettsii* qPCR assay depicted conservation within all available *R*. *rickettsii* gene sequences, a lack of gene similarity in whole genome DNA analysis of other *Rickettsia* species, and the absence of amplification of the available near-neighbor *R*. *typhi* reference control (Supplementary Fig. S1I). Further studies should be conducted to further verify assay specificity in respect to additional *Rickettsia* species reference controls.

*In silico* analysis limitations also extend to the *Anaplasma phagocytophilum* and *Borrelia turicatae* assays and their respective targeted *msp2* and *bipA* genes. As in the case of *R*. *rickettsii*, limited *msp2* gene sequences are available due to pathogen specific conservation^22^. However, the *bipA* gene has been shown to be conserved in other tick-borne relapsing fever (TBRF) species, although a majority of these species do not have *bipA* sequences available in NCBI^35^. Nevertheless, despite limited *in silico* analysis of both A. *phagocytophilum* and *B*. *turicatae*, qPCR analysis against numerous close neighbor reference controls supports assay specificity.

With respect to *in silico* analysis of the *Borrelia burgdorferi* assay, there are numerous mismatches in which the oligonucleotides are situated in comparison to gene sequences from various species within the *B*. *burgdorferi sensu lato* complex (BBSL) (Supplementary Fig. S1A). The BBSL group, at present, contains roughly 20 identified spirochete species with public health concern^36^. However, due to limited reference control availability it is not conclusive if the assay will not cross-amplify and subsequently detect these species during qPCR screening. Therefore, future studies should be conducted to verify the specificity of the assay to *B*. *burgdorferi sensu stricto* when screening samples with unknown BBSL infection status.

This study, despite the aforementioned considerations, achieved the goal of simultaneously screening for 11 targets through layerplex methodology. The assay has also demonstrated effectiveness as a valuable surveillance assay^5,37,38^, and molecular diagnostic tool (https://tvmdl.tamu.edu/tests/tickpath-layerplex-qpcr/). Collectively, these findings provide support for additional studies to further validate the layerplex qPCR capabilities in terms of applying the methodology to different disease groups that require the detection of a broad spectrum of pathogens. Further studies should also assess the limits of detection for the technology with multiple targets labeled in a given reaction. In conclusion, this evaluation demonstrated the capacity of the TickPath Layerplex assay to detect *Borrelia hermsii*, *B*. *turicatae*, *B*. *parkeri*, *B*. *burgdorferi*, *Ehrlichia canis*, *E*. *chaffeensis*, *E*. *ewingii*, *Anaplasma phagocytophilum*, *Rickettsia rickettsii*, and *Babesia* species DNA simultaneously with high sensitivity and specificity in clinical domesticated dog and tick specimens.

## Methods

### Reference controls

Bacteria and protozoa strains used for the analytical sensitivity and specificity analysis were as follows. Culture from wildlife isolates: *Borrelia hermsii* GGI DAH, *B*. *hermsii* GGII MTW-4, *B*. *turicatae* TCBP2, *B*. *parkeri* SLO, *B*. *miyamotoi* FR64b, *B*. *coriaceae* Co53, *B*. *anserina* BA-2, *B*. *crocidurae* DOS-56, and *B*. *recurrentis* 132 were made available by Dr. Tom Schwann at the Laboratory of Zoonotic Pathogens Rocky Mountain Laboratory NIAID Facility. Laboratory isolates: *Borrelia burgdorferi* B31 MSK5, *B*. *burgdorferi* B31 A3, *B*. *garinii* (ATCC® 5183^TM^), and *B*. *afzelii* (ATCC® 51567) were provided by Dr. Maria Esteve-Gasent. Laboratory isolates: *Babesia conradae* Wideload, *B*. *microti* CMNI, and *B*. *duncani* COA3 were cultured and supplied by Dr. Patricia Conrad at UC Davis School of Veterinary Medicine. Laboratory isolates: *Babesia canis vogeli* Buster, *Babesia gibsoni* Ruby, *Babesia duncani* WA3, *B*. *bovis* Mexico, *B*. *bovis* TAMU, *B*. *caballi* Mexico, *B*. *caballi* USDA, *B*. *divergens* Purnell, *B*. *microti* Ruebush, *B*. *odocoilei* Wisconsin, *B*. *bigemina*, *Theileria equi* USDA, and *Cytauxzoon felis* Tyson were prepared by Dr. Patricia Holman at the Texas A&M College of Veterinary Medicine. Genomic DNA isolated from FA substrate slides (VMRD, Pullman, WA; Focus Diagnostics, Cypress, CA; Protatek, St. Paul, MN): *Ehrlichia canis* (SLD-IFA-EC), *E*. *chaffeensis* (IF1003), *Anaplasma phagocytophilum* (AG-112), *Rickettsia rickettsii* (SLD-IFA-RMSF), *R*. *typhi* (SLD-IFA-RMSF), *Babesia canis* (AG-119), *B*. *gibsoni* (AG-123), and *Borrelia burgdorferi* (SLD-IFA-LD). Substrate slides were prepared for DNA extraction by rehydration with 10.0 μL Phosphate-buffered saline (PBS) onto a single slide well and then suspended in 90.0 μL PBS. The 100.0 μL solution was then purified using the MagMAX^TM^ Nucleic Acid Isolation Kit AMB1836 (Thermo Fisher Scientific, Waltham, MA) following manufacturers recommendations adopted from a previous publication^39^. *16S rRNA* gene gBlocks (IDT, San Jose CA) consisting of: *Ehrlichia ewingii* Stillwater (NR_044747, 1435 base pairs), *E*. *ruminantium* Welgevonden (NR_074513, 1507 base pairs), *E*. *muris* AS145 (NR_025962, 1428 base pairs). gBlocks were utilized for validation purposes due to the inability to obtain genomic DNA for the aforementioned isolates. The artificially synthesized gBlocks were constructed based on respective pathogen type-strain *16S rRNA* sequences found in the National Center for Biotechnology Information (NCBI) database. Genomic DNA reference strains provided by the Texas A&M Veterinary Medical Diagnostic Laboratory (TVMDL): *Ehrlichia canis*, *Anaplasma phagocytophilum*, *A*. *marginale*, *A*. *centrale*, and *A*. *ovis*.

### Clinical and diagnostic sample collection

In addition to tick vectors, the optimal pre-mortem biological sample type for screening the aforementioned tick-borne pathogens in dogs is whole blood^4,40–42^. DNA detection of Bb within blood is considered rare and generally inadvisable, though studies have documented a low percentage of detection of the spirochete in blood samples^17,43^. Therefore, the majority of validation analysis for the assay detailed in this study focused on utilizing ticks and whole blood samples. However, due to the limited blood borne circulation nature of the Bb spirochete, laboratory infected mice were utilized to simulate infection. 9 infected skin biopsies and 9 joints were harvested for analysis. This study also involved the use of 1,171 archived blood samples originating from dogs that were submitted to two full-service Texas A&M Veterinary Service Laboratory (TVMDL) facilities (i.e. College Station, TX and Amarillo, TX). All samples were archived after the 15-day hold period in which TVMDL analyzed the samples and provided results to veterinarians, but prior to their destruction and disposal. Further, a total of 211 *Rhipicephalus sanguineus* (brown dog ticks), and 35 *Ixodes scapularis* (black-legged ticks) were utilized for this study.

### Animal care

All mice related procedures were performed and approved in accordance with the Institutional Biosafety Committee (IBC 2016-051) and the Institutional Animal Care and Use Committee (IACUC) at Texas A&M University as detailed in the Animal Use Protocol (AUP 2017-0022). The mice were maintained in an animal facility accredited by the Association for Assessment and Accreditation of Laboratory Animal Care (AAALAC). Dog samples screened in this study were reviewed by the IBC and IACUC, and considered “surplus blood” exempt of permit. Dogs were not recruited for this study, and no direct handling of dogs was done by the research team.

### Oligonucleotide design

Pathogens were separated into three groups (layers) based on species classification: borrelial (*Borrelia* species: Bb, Bh, Bt, Bp), rickettsial (Rickettsiales pathogens: Rr, Ap, Ec, Ech, Ee), and babesial (*Babesia* species: Bab). Assays were designed manually to target specific gene targets (i.e. *16S rRNA* for *Ehrlichia* spp., *msp2* for *Anaplasma phagocytophilum*, *18S rRNA* for *Babesia* spp., *flaB* and *bipA* for *Borrelia* spp., and *rrhyp* for *Rickettsia rickettsii*) based on previous studies^19,22,33,43–46^. Up to 100 individual nucleotide sequences available in GenBank^®^ greater than 500 bp for each target pathogen were evaluated with gene sequences from closely related species for each respective layer as detailed in the supplementary information (n = 610, 70, 564, 268, 86, and 12 for *16S rRNA*, *msp2*, *18S rRNA*, *flaB*, *bipA*, and *rrhyp*, respectively; Supplementary Tables S5–10) as demonstrated previously^33^. Due to the conservation of the *msp2* and *rrhyp* genes within the respective pathogens, available sequences were limited to only target pathogens for evaluation. CLC Main Workbench 7.7 (CLCbio, Aarhus, Denmark), Primer Express 3.0 software (Thermo Fisher Scientific, Waltham, MA), and NCBI BLAST^®^ were used for oligonucleotide selection and evaluation as previously described^33,47^. Primers and probes were manually positioned over regions featuring high conservation and dissimilarity in regards to homologous and heterologous gene sequences, respectively. Sequences were then assessed on the basis of previously established criteria, and mismatch locations in respect to heterologous pathogens were identified^32^. Final reaction concentrations and sequence information for the oligonucleotides utilized in the qPCR assay are provided in Table 1. All primers and Dual-Labeled BHQ® Probes were acquired commercially (LGC Biosearch Technologies, Petaluma, CA). Primer and probe sequences for detection of Bb, Bh, Bt, Bp, Ec, Ech, Ee, Ap, Rr, and Bab were developed in house, EIPC-K9 was taken from a previous publication, and optimal oligonucleotide concentrations were established through empirical testing^32^.

### Plasmid positive amplification control (PAC) DNA

A single plasmid control featuring the Bb, Bh, Bt, Bp, Ec, Ech, Ee, Ap, Rr, and Bab target regions was employed as a PAC, and to calculate the analytical sensitivity and limit of detection (LOD). The plasmid PAC was synthetically prepared as demonstrated previously^33^. The purified plasmid was quantified using a NanoDrop™ 8000 Spectrophotometer (Thermo Fisher Scientific, Waltham, MA). An EIPC-K9 plasmid was prepared as previously described^32^. The final PAC was prepared by combining the target pathogen plasmid DNA with EIPC-K9 target plasmid DNA at 1,000 and 150,000 copies/μL, respectively. The PAC was used for each qPCR run to ensure consistent qPCR conditions.

### Nucleic acid purification

Nucleic acid purification performed on all samples used in this study for assay validation utilized the MagMAX^TM^ Nucleic Acid Isolation Kit AMB1836 (Thermo Fisher Scientific, Waltham, MA) following manufacturers recommendations adopted from a previous publication^39^.

### Layerplex real-time PCR

In order to facilitate the simultaneous screening off all pathogens in a single multiplex format, multiple pathogens were grouped together under specific “layers” and labeled with the same Dual-Labeled BHQ® Probe. Layers were created based on species similarity and labeled with the same fluorogenic probe: borrelial: Bh, Bt, Bp, Bb; rickettsial: Ec, Ech, Ee, Ap, Rr; and pan-*Babesia* species babesial: Bab. A fourth probe was also utilized in order to label an 11^th^ target, represented as an endogenous internal positive control for diagnostic quality control of DNA extractions^32^. Termed here as “layerplexing”, four unique probes were utilized to accommodate all 10 assays and the EIPC-K9 in a single qPCR reaction. The layerplex qPCR was performed with Bb, Bh, Bt, Bp, Ec, Ech, Ee, Ap, Rr, Bab, and EIPC-K9 specific primers and probes (Table 1) using qPCR Path-ID^TM^ buffer (Thermo Fisher Scientific, Waltham, MA) according to manufacturer’s instructions. Each 25 μL of qPCR contained 12.5 μL of 2x qPCR buffer, 2 μL of 12.5x primer-probe mix consisting of all oligonucleotides in Table 1, 2.5 μL of nuclease-free water, and 8 μL of nucleic acid template. Layerplex qPCR was performed using an Applied Biosystems® 7500 Fast Real-Time PCR System (Thermo Fisher Scientific). Cycling conditions (thermal profile) consisted of an initial denaturation and activation at 95 °C for 10 min, and 40 cycles of amplification at 95 °C for 1 sec and 60 °C for 30 sec, for a total run time of 59 min. Samples with a quantification cycle (Cq) ≤38 were considered positive.

### Singleplex and conventional multiplex real-time PCR

Singleplex analyses to detect individual pathogens were conducted by following the same qPCR reaction template with modifications to the primer-probe mixes. For specific species detection, the associated primer-probe mix was added in place of the layerplex primer-probe mix and was dependent upon intended species detection as detailed further in Supplementary Fig. S1. Conventional multiplex qPCR analysis where each probe labels only one species assay is feasible for a combination of most pathogens, including *Borrelia* spp. and Rickettsiales pathogens. However, as the *Ehrlichia* spp. share a probe label, singleplex analysis would be required if an *Ehrlichia* spp. infection is suspected. Future studies should be conducted in order to further validate individual use of the multiplex capabilities.

### Confirmatory conventional PCR analysis

Conventional PCR analysis was utilized to verify all positive and suspect results from layerplex qPCR analysis. Conventional PCR protocols for the detection of the *16S rRNA* gene of *Ehrlichia* and *Anaplasma* species, the *ompA* gene of *Rickettsia* species, the intergenic spacer sequence (*16S rRNA–23S rRNA*) of *Borrelia* species, and the *18S rRNA* of *Babesia* species were utilized as described previously^16,48–50^. Positive (genomic) controls and negative controls (water) were included in all PCR assays. Sanger sequencing (Eurofins Scientific, Louisville, KY) in both directions was used to obtain a consensus sequence from all attained DNA amplicons.

### Data analysis

Efficiency and R^2^ values of the polymerase chain reactions was determined using the methods described previously^51,52^. To determine the LOD in terms of copy number, linear regression analysis and serial dilutions of the PAC (working stock: 7.59 × 10^−5^ ng/μL or 1.27 × 10^5^ copies/μL) were used as template. The LOD was conveyed as copy number of the plasmid per assay where each copy of the plasmid represents 1 copy of the pathogen’s genome, or genome copy equivalents per microliter (GCE/μL). The copy number of the plasmid was determined using the formula described previously^51^. Diagnostic test evaluation for determining sensitivity and specificity, with 95% confidence intervals, was determined by following previously established formulas^53^.

## Supplementary information

Supplementary Figures

Supplementary Tables

## Data Availability

All data generated during this study is included in this article. Other relevant data supporting the findings of the study are available in the Supplementary Information Files.
