# Supplementary material for: TickPath Layerplex: adaptation of a real-time PCR methodology for the simultaneous detection and molecular surveillance of tick-borne pathogens
Source: Sci Rep. 2019 May 6;9:6950. doi: 10.1038/s41598-019-43424-y (PMC6502835; doi:10.1038/s41598-019-43424-y)
Supplement: Supplementary file 1 — Supplementary Figures [file 41598_2019_43424_MOESM1_ESM.pdf]

TITLE:

TickPath Layerplex: adaptation of a real-time PCR methodology for the simultaneous detection and molecular surveillance of tick-borne pathogens

AUTHORS:

Joseph J. Modarelli[1], Pamela J. Ferro[2], Alberto A. Perez De Leon[3], Maria D. Esteve-Gasent[1]*

AFFILIATION:

[1] Department of Veterinary Pathobiology, College of Veterinary Medicine and Biomedical Sciences, Texas A&M University, College Station, TX, 77843.

[2] Texas A&M Veterinary Medical Diagnostic Laboratory, Texas A&M University, College Station, TX, 77843

[3]USDA–ARS Knipling–Bushland U.S. Livestock Insects Research Laboratory and Veterinary Pest Genomics Center, Kerrville, TX, 78028.

*Correspondence and requests for materials should be addressed to M.E.G. (email: MEsteve-Gassent@cvm.tamu.edu)

Supplementary Figure S1. Singleplex *in silico* specificity analysis depicted by oligonucleotide alignments for the forward primer, reverse primer, and probe sequences of (A) *B. burgdorferi*, (B) *B. hermsii*, (C) *B. parkeri*, (D) *B. turicatae*, (E) *E. ewingii*, (F) *E. chaffeensis*, (G) *E. canis*, (H) *A. phagocytophilum*, (I) *R. rickettsii*, and (J) pan-*Babesia* assays in 5' to 3' orientation. Assays were evaluated for analytical specificity against representative closely related species as indicated in Supplementary Tables S5-S10. Respective primers and probes used for analysis are highlighted in grey. The sequences for other species are only shown when they differ from respective species sequences. A dot indicates identity with the respective species sequence. Dashes in (A)-(C), (E)-(G) and (J) refer to an in/del, dashes in (D), (H) and (I) refer to absence of gene sequences. The second column indicates specificity in qualitative results (positive or negative). An asterisk represents hypothetical analysis based on NCBI BLAST analysis due to lack of available reference strains.

(A)

Organism	qPCR	Bb.flaB.161F (5' → 3')	Bb.flaB.186P (5' → 3')	Bb.flaB.213R (5' → 3')
B. burgdorferi	+	A A G A G G G T G T T C A A C A G G A A G G	T C A A C A G - - - C C A G C A C C T G C T A C A G C A	G A G A A T T A A C T C C G C C T T G A G A A
B. garinii	-	. . . . A . . . . C . . . . . . A	. . . . . . A . . . . G	. . . . A . . . .
B. afzelii	-	. . . . C . . . . G . A	. . . G . . A . . . . . . A	. . . . A . . . .
B. anserina	-	. . . . A . C A . . . . . A . . G . .	G . . . G C A A C T . . . . . T . . A . T A G . T . . T	. . . . C . . A . . . G . . . C T
B. miyamotoi	-	. . . . A . C A . . . . G . .	. . . . G C A G C T . . . . . T . . A . . A G . C . . T	. . . . A . . . . C T
B. recurrentis	-	. . . . C A . . G . . A	A . . . G C A G C T . . . . . T . . A . . A G . . . . T	. . . . G . . . . A . . . . C T
B. crocidurae	-	. . . . C A . . G . . A	A . . . G C A G C T . . . . A . . A G . . . . T	. . . . G . . . . A . . . . C T
B. turicatae	-	. G . . A . . . . C A . . G . . A . . G . .	. . . . G C T G C T . . . . A . . A G . . . . T	. . . . A . . . . C T
B. parkeri	-	. G . . A . . . . C A . . . . . A . . G . .	. . . . G C T G C T . . . . A . . A G . . . . T	. T . . . . A . . . . C T
B. hermsii GGI	-	. . . . . A T A . G A . . G . . A . . G . A	. . . . G C A G C T . . . . . T . . A . . A G . . . . T	. G . . . . A . . . . C T
B. hermsii GGII	-	. . . . . A T A . G A . . G . . A . . G . A	. . . . G C A G C T . . . . . T . . A . . A G . . . . T	. . . . C . . A . . . . C T
B. coriaceae	-	C T . . A . . . . C A . . . G . A	. . . . G C T G G A . A . . G T G . A . T A G . . . . T	. . . . A . . . . C T

(B)

Organism	qPCR	Bh-1.flaB.531F / Bh-1.flaB.529F (5' → 3')	Bh.flaB.615P (5' → 3')	Bh-1.flaB.647R (5' → 3')
B. hermsii GGI	+	. . G G G C G C A A A T C A G G A T G A G	A G C - - - - - - - - - C T G A G C G C C T T C A C C T G C A A A A A G A	T C C T C T T G C T G T C C T A T C T C T T G C
B. hermsii GGII	+	G T G G G A G C A A A T C A G G A T G A G	. . . .	. . . .
B. turicatae	-	. . . . . T . . . . A	. A . . . . C . . A . . . . G	C . . . . T . . . G . A C C T . . C . G A
B. parkeri	-	. . . . . T . . . . A	. A . . . . C . . A . . . . G	C . . . . T . . . G . A C C T . . C . G A
B. miyamotoi	-	. . A . . T . . . . A	T . . . . T . . . . . A . . . . A T T	C . . . . C . . T . . . G . . C C . . . . . G A
B. coriaceae	-	. . . . C . . A	. . G . . . . A	C . T . . . . C T . . . G . A C C T . . - - - A
B. anserina	-	. . . . . T . . . . A	. . . . A	C . . . . T . . . G . . C C . . . . . . A
B. crocidurae	-	. . T . . T . . . . A	T A . T T G . . . . A . . C . . . . A	C . T . . . . G . A C C . . . . . . A
B. recurrentis	-	. . T . . T . . . . A	T A . T T G . . . . . . T . . . . . A . . C . . . . A	C . T . . . . G . A C C . . . . . . A
B. burgdorferi	-	. . T . . . . C . . A . . . . . A	. . . C T G A G C A G T T . . . . . T . . C . . . . . A . A G	C . T . . C . . T . . A A . A C C . . . . . . A
B. afzelii	-	. . . . A . . . . . A	. . . C T G A G C A G C T . . . . . T . . C . . . . . A	C . T . . . . C T . . A G . A C C . . . . . . A
B. garinii	-	. . . . A . . . . . A	. . T C T G A G C A G C . . . . . . T . . . . . G . . A . A G . . T . . .	C . T . . . . . T . . A G . A C C T . . . . . A

(C)

Organism	qPCR	Bp.flaB.406F (5' → 3')	Bp.flaB.443P (5' → 3')	Bp.flaB.522R (5' → 3')
B. parkeri	+	TTGTCCAATAAGTCAGCTGCTCAG	CTGAAGAGCTTGGAATGCAACCTGCA	TCTTAATGTCCATGAAGCTTGTGC
B. turicatae	-	........C..............A	....	.......................A
B. hermsii GGI	-	........C..............A	....	...C...................A
B. hermsii GGII	-	........C..............A	....	...C...................A
B. anserina	-	.....T..C..............A	...................G......	.......................A
B. coriaceae	-	.....T.....A.........G..	....	...C...................A
B. miyamotoi	-	..A..T.................A	....	...C....................
B. recurrentis	-	..A..T....GA...T.....G..	........................T.	.......................A
B. crocidurae	-	..A..T....GA...T.....G.A	........................T.	.......................A
B. burgdorferi	-	..A..A..C..A..T...T....A	...................G......	......A.....A..C.....A.A
B. afzelii	-	.....A..C..A..T...T.C..A	...................G......	......A.....A........A.A
B. garinii	-	.....A..C..A..T...T.C..A	.......A..................	......G.....A........A.A

(D)

Organsim	qPCR	Bt.bipA.728F / Bt.bipA.731F (5' → 3')	Bt.bipA.817P (5' → 3')	Bt.bipA.869R (5' → 3')
B. turicatae 1	+	AGACCGGTACACAGGATTCTAAAGC	AGTTTTGGGAAGTGTTGTTGGTGGCGT	GTTCCTGCTCCCTGAATAACATTATC
B. turicatae 2	+	...CCGGCACACAGGATTCTAAAAC	....	....
B. parkeri	-	.T..TAAA................A	.A.........A...............	....
B. hermsii GGI	-	..TTC.C.GTC.T..G.A.AGT..A	.A..GA.TATG.AC...AGAA...GA.	..CT.........TGC..G.GG.C.T
B. hermsii GGII	-	..TTT.C.GTTTT..G.A.AGT..A	.A..GA.MATG.AC...AGAA...GAC	..C.........CT.C..G.GGCC.T
B. miyamotoi	-	-------------------------	---------------------------	--------------------------
B. coriaceae	-	-------------------------	---------------------------	--------------------------
B. anserina	-	-------------------------	---------------------------	--------------------------
B. crocidurae	-	-------------------------	---------------------------	--------------------------
B. recurrentis	-	-------------------------	---------------------------	--------------------------
B. burgdorferi	-	-------------------------	---------------------------	--------------------------
B. afzelii	-	-------------------------	---------------------------	--------------------------
B. garinii	-	-------------------------	---------------------------	--------------------------

(E)

Organism	qPCR	Ee.16S.40F (5' → 3')	Ehr-spp.16S.83P (5' → 3')	Ech-ee.16S.174R (5' → 3')
E. ewingii	+	CGAACGAACAATTCCTAAATAGTCTCT	AGATTCCTACGCATTACTCACCCGTCTGC	CCATCATTTCTAATGGCTATTCCATACT
E. canis	-	......G......AT.T.TAGCCTCTG	....	...C...................G....
E. muris	-	......G.T.GC.A.CC.TAGC.T.T.	.........T...................	....
E. ruminantium	-	......G...G..AT.T.TAGC.TCGG	....	.T............A.............
E. chaffeensis	-	......G......G..T.TA.CCT.T.	....	....
A. phagocytophilum	-	......G.TT....T.T.TAGC.TG..	.........T...................	...C........G.......C.......
A. marginale	-	......G..CG-.ATACGCAGC.TG..	.........T...................	...C........G.......C.......
A. ovis	-	......G..CG-.A.GCGCAGC.TG..	.........T...................	...C........G.......C.T.....
A. centrale	-	......G..C.-.A.GCGCAGC.TG..	.........T...................	...C........G.......C.T.....
A. bovis	-	.........T.....-C.TAGC.WG..	.........T...................	...C..C.....G.......C.T.....
R. rickettsii	-	.A....G.ACGC.ATCGGTAT.CT.AA	.......C...TG................	.TT.T........AA.T......G....
R. typhi	-	.AG.ACG.ACGC.ATCGGTAT.CT.AA	.......C...TG................	.TT.T........AA.T......G....

(F)

Organism	qPCR	Ech.16S.64F (5' → 3')	Ehr-spp.16S.83P (5' → 3')	Ech-ee.16S.174R (5' → 3')
E. chaffeensis	+	GAACGGACAATTGCTTATAACCTTTT	AGATTCCTACGCATTACTCACCCGTCTGC	CCATCATTTCTAATGGCTATTCCATACT
E. canis	-	............AT.....G...C.G	....	...C...................G....
E. muris	-	.......T.GC.A.CC...G.T....	.........T...................	....
E. ruminantium	-	.........G..AT.....G.T.CGG	....	.T............A.............
E. ewingii	-	.....A......C..A.AT.GTC.C.	....	....
A. phagocytophilum	-	.......TT...CT.....G.T.GC.	.........T...................	...C........G.......C.......
A. marginale	-	........CG-.ATACGC.G.T.GC.	.........T...................	...C........G.......C.......
A. ovis	-	........CG-.A.GCGC.G.T.GC.	.........T...................	...C........G.......C.T.....
A. centrale	-	........C.-.A.GCGC.G.T.GC.	.........T...................	...C........G.......C.T.....
A. bovis	-	........T...-.CC...G.W.GC.	.........T...................	...C..C.....G.......C.T.....
R. rickettsii	-	A......ACGC.ATCGG..TG...AA	.......C...TG................	.TT.T........AA.T......G....
R. typhi	-	AG.AC..ACGC.ATCGG..TG...AA	.......C...TG................	.TT.T........AA.T......G....

(G)

Organism	qPCR	Ec.16S.61F (5' → 3')	Ehr-spp.16S.83P (5' → 3')	Ec.16S.148R (5' → 3')
E. canis	+	G C C T C T - - G G C T A T A G G - A A A T T G T T A G T	A G A T T C C T A C G C A T T A C T C A C C C G T C T G C	C T C G G G G A T T A T A C A G T A T T A C C C A C
E. chaffeensis	-	A . . . T . T . . . T . . . . A A T . -	. . . .	. G . A . . . . T
E. muris	-	. . T . T . T T A . . . . T T C G C . A	. . . . T	. G . A . . . . T
E. ruminantium	-	. . T . . - . . . . G A . T . T - C	. . . .	. G . A . . . . T . T
E. ewingii	-	A G T C T C T G A C T A T . T A . A T . G	. . . .	. G . A . . . . T
A. phagocytophilum	-	. . T . - - . . - . . . . . G . A G G . T A - A	. . . . T	. G . A . . . .
A. marginale	-	. . T . G C . . - - - - - . G C . T G T . . G	. . . . T	. G . A . . . .
A. ovis	-	. . T . G C . . - - - - - . G C . T G T . . G	. . . . T	. G . A . . . .
A. centrale	-	. . T . G C . . - - - - - . G C . T G T . . G	. . . . T	. G . A . . . .
A. bovis	-	. . W . G . . . - - - - - . . T . G G . . . A	. . . . T	. G . A . . . .
R. rickettsii	-	. T A A T . G G . . . . T G C T C C . . T . A	. . . . C . . . T G	. G . A . A . . A . . . . . G . . . . . . G . T T T
R. typhi	-	T T A A T . A G A . . . T G C T C T . G T . A A	. . . . C . . . T G	. G T A . A . A A . . . . . G . . . . . . G . T T T

(H)

Organism	qPCR	Ap.msp2.420F (5' → 3')	Ap.msp2.452P (5' → 3')	Ap.msp2.514R (5' → 3')
A. phagocytophilum	+	G A C T T T C C T A G C A T G G A G T T G G T T	C A T T T C A C C T T A C A C A T G C G C C G G A	G C G T G C C C T T T T G T A A T A C C T A T A A
A. marginale	-	- -	- -	- -
A. ovis	-	- -	- -	- -
A. centrale	-	- -	- -	- -
A. bovis	-	- -	- -	- -
E. chaffeensis	-	- -	- -	- -
E. canis	-	- -	- -	- -
E. muris	-	- -	- -	- -
E. ruminantium	-	- -	- -	- -
E. ewingii	-	- -	- -	- -
R. rickettsii	-	- -	- -	- -
R. typhi	-	- -	- -	- -

(I)

Organism	qPCR	Rr.hyp.724702F (5' → 3')	Rr.hyp.724788P (5' → 3')	Rr.hyp.724860R (5' → 3')
R. rickettsii	+	A G A G T A A A T C A A C G G A A G A G C A A A A C	T C C T C T C C A A T C A G C G A T T C A G G C A	C C C C T C C A C T A C C T G C A T C A T
R. typhi	-	- -	- -	- -
A. phagocytophilum	-	- -	- -	- -
A. marginale	-	- -	- -	- -
A. ovis	-	- -	- -	- -
A. centrale	-	- -	- -	- -
A. bovis	-	- -	- -	- -
E. chaffeensis	-	- -	- -	- -
E. canis	-	- -	- -	- -
E. muris	-	- -	- -	- -
E. ruminantium	-	- -	- -	- -
E. ewingii	-	- -	- -	- -
R. akari	-*	- -	- -	- -
R. amblyommii	-*	- -	- -	- -
R. australis	-*	- -	- -	- -
R. canadensis	-*	- -	- -	- -
R. conorii	-*	- -	- -	- -
R. felis	-*	- -	- -	- -
R. honei	-*	- -	- -	- -
R. massiliae	-*	- -	- -	- -
R. montana	-*	- -	- -	- -
R. rhipicephali	-*	- -	- -	- -
R. parkeri	-*	- -	- -	- -
R. sibirica	-*	- -	- -	- -
R. slovaca	-*	- -	- -	- -

(J)

Organism	qPCR	Babsp.18S.65-1F / Babsp.18S.67-2F (5' → 3')	Babsp.18S.228P (5' → 3')	Babsp.18S.289R (5' → 3')
B. canis vogeli	+	C G C A - - - - T T T A - - - - G C G A T G G A C C A	C A T C A G C T T - - G A C G G T A G G G T A T T G G C C	C C T A A T T C C C C G T T A C C C G T T
B. canis rossi	+	. G C - - - - T T T T A - - - - G C G A T G G A C C A T T C A	. . . .	. . . .
B. canis canis	+	. . . .	. . . .	. . . .
B. gibsoni	+	. . . .	. . . .	. . . .
B. caballi	+	. . . . . . T . . . . C	. . . .	. . . .
B. odocoilei	+	. . . . A . T . . A . T	. . . .	. . . .
B. divergens	+	. . . . A . T . . . . T	. . . .	. . . .
B. bigemina	+	. . . . T . . . . T T	. . . .	. . . .
B. bovis	-	. . . . T . . . . A T T	. . . .	 C
B. microti	-	. . . . T G G C . . . - G C C G . . . . . . T . T	T . . . . T G	. . C . . . . C C
B. duncani	-	. . . . T G G C . . . T G C C G . . . . T T	T . . . . T G	 C
B. conradae	-	. . . . T G G C . . . T G C C G . . . . . A A T T	T . . . . T G	 C
T. eqeui	-	. . . . T G G C . . . T G C T G . . . . . . T . T	T . . . . T G	 C
C. felis	-	. . . . T G G C . . . - G C C G . . . . . . T . T	T . . . . T G	 C
