# Supplementary material for: TickPath Layerplex: adaptation of a real-time PCR methodology for the simultaneous detection and molecular surveillance of tick-borne pathogens
Source: Sci Rep. 2019 May 6;9:6950. doi: 10.1038/s41598-019-43424-y (PMC6502835; doi:10.1038/s41598-019-43424-y)
Supplement: Supplementary file 2 — Supplementary Tables [file 41598_2019_43424_MOESM2_ESM.pdf]

TITLE:

TickPath Layerplex: adaptation of a real-time PCR methodology for the simultaneous detection and molecular surveillance of tick-borne pathogens

AUTHORS:

Joseph J. Modarelli[1], Pamela J. Ferro[2], Alberto A. Perez De Leon[3], Maria D. Esteve-Gasent[1]*

AFFILIATION:

[1] Department of Veterinary Pathobiology, College of Veterinary Medicine and Biomedical Sciences, Texas A&M University, College Station, TX, 77843.

[2] Texas A&M Veterinary Medical Diagnostic Laboratory, Texas A&M University, College Station, TX, 77843.

[3]USDA–ARS Knipling–Bushland U.S. Livestock Insects Research Laboratory and Veterinary Pest Genomics Center, Kerrville, TX, 78028.

*Correspondence and requests for materials should be addressed to M.E.G. (email: MEsteve-Gassent@cvm.tamu.edu)

Supplementary Table S1. Limit of detection analysis of the *Borrelia* species assays. Quantification cycle (Cq) values reported in mean from duplicate testing. Plasmid DNA concentrations not tested are represented by NA.

Fold	Plasmid DNA (ng/µl)	Target Copy #	*B. hermsii* Cq	*B. burgdorferi* Cq	*B. parkeri* Cq	*B. turicatae* Cq
1	75.91	1.27E+11	NA	NA	NA	NA
1.00E-06	7.59E-05	127200	21.3	21.3	22.4	21.4
1.00E-07	7.59E-06	12720	24.6	24.6	25.8	24.9
1.00E-08	7.59E-07	1272	27.8	27.8	29.0	28.1
1.00E-09	7.59E-08	127	30.9	30.9	32.4	31.5
1.00E-10	7.59E-09	13	33.9	35.3	36.3	35.0
5.00E-11	3.80E-09	6	36.1	36.0	36.5	36.7
2.50E-11	1.90E-09	3	36.8	36.4	37.7	35.6

Supplementary Table S2. Limit of detection analysis of the *Ehrlichia, Anaplasma, and Rickettsia* species assays. Quantification cycle (Cq) values reported in mean from duplicate testing. Plasmid DNA concentrations not tested are represented by NA.

Fold	Plasmid DNA (ng/µl)	Target Copy #	*E. canis* Cq	*E. chaffeensis* Cq	*E. ewingii* Cq	*A. phagocytophilum* Cq	*R. rickettsii* Cq
1	75.91	1.27E+11	NA	NA	NA	NA	NA
1.00E-06	7.59E-05	127,200	22.1	23.6	23.2	21.8	22.1
1.00E-07	7.59E-06	12,720	25.4	27.1	26.6	25.2	25.5
1.00E-08	7.59E-07	1,272	28.5	30.3	30.1	28.3	28.8
1.00E-09	7.59E-08	127	31.9	34.0	33.7	31.9	32.2
2.50E-10	1.90E-08	32	34.8	36.5	35.8	34.7	33.7
1.25E-10	9.49E-09	16	35.1	36.7	37.1	34.8	35.9

Supplementary Table S3. Limit of detection analysis of the *Babesia* species assays. Quantification cycle (Cq) values reported in mean from duplicate testing. Plasmid DNA concentrations not tested are represented by NA.

Fold	Plasmid DNA (ng/µl)	Target Copy #	*Babesia* spp. Cq
1	75.91	1.27E+11	NA
1.00E-06	7.59E-05	127,200	21.8
1.00E-07	7.59E-06	12,720	24.6
1.00E-08	7.59E-07	1,272	28.0
1.00E-09	7.59E-08	127	32.0
2.50E-10	1.90E-08	32	34.2
1.25E-10	9.49E-09	16	35.6

Supplementary Table S4. Limit of detection analysis of the Borrelial, Rickettsial, and Babesial layers evaluated in layerplex format. Quantification cycle (Cq) values reported in mean from duplicate testing. Plasmid DNA concentrations not tested are represented by NA.

Fold	Plasmid DNA (ng/µl)	Target Copy #	Borrelial Cq	Rickettsial Cq	Babesial Cq
1	75.91	1.27E+11	NA	NA	NA
1.00E-06	7.59E-05	127,200	23.2	23.0	22.1
1.00E-07	7.59E-06	12,720	26.0	25.7	24.8
1.00E-08	7.59E-07	1,272	29.1	29.0	28.1
1.00E-09	7.59E-08	127	32.8	32.5	31.5
2.50E-10	1.90E-08	32	34.6	34.4	33.5
1.25E-10	9.49E-09	16	35.5	35.4	34.4

Supplementary Table S5. GenBank® accession codes for the *16S rRNA* gene sequences of *Ehrlichia*, *Anaplasma,* and *Rickettsia* species. Sequences utilized for *in silico* analysis in Supplementary Fig. S1 are emphasized in bold.

Rickettsial Species	Isolate/Strain	GeneBank®
E. canis	NA	U26740
E. canis	GR78	EF011111
E. canis	GR21	EF011110
E. canis	Kiwi	HQ844983
E. canis	TWN1	EU106856
E. canis	Kagoshima	AF536827
E. canis	NA	KM879929
E. canis	171	KC479023
E. canis	105	KC479022
E. canis	MSIA	JF429693
E. canis	B	JX893523
E. canis	A	JX893522
E. canis	NGR	JN982339
E. canis	NGR	JN982336
E. canis	NGR	JN622141
E. canis	TWN4	EU143637
E. canis	Nero	EU439944
E. canis	TWN	GU810149
E. canis	TWN3	EU143636
E. canis	TWN2	EU123923
E. canis	DT	HQ290362
E. canis	NA	EF139458
E. canis	TWN17	EU139493
E. canis	Kutahya	AY621071
E. canis	TWN18	EU178797
E. canis	NA	DQ915970
E. canis	NA	AY394465
E. canis	VDE	AF373613
E. canis	VHE	AF373612
E. canis	Gxht67	AF156786
E. canis	Gdt3	AF156785
E. canis	b2-15	KY594915
E. canis	WHBMXZ-124	KX987326
E. canis	M66	KX180945
E. canis	NA	KR920044
E. canis	**Oklahoma**	**NR_118741**
E. canis	S3b	KJ659037
E. canis	TrKysEcan3	KJ513197
E. canis	TrKysEcan2	KJ513196
E. canis	TrKysEcan1	KJ513194
E. canis	CMM-19-2002	AB723711
E. canis	CMM-19-2006	AB723712
E. canis	W-137J	AB723710
E. canis	E-60	AB723709
E. canis	W-134	AB723708
E. canis	E-89J	AB723707
E. canis	Bareilly	JX861392
E. canis	Brazil-CO2	EF195135
E. canis	Brazil-CO1	EF195134
E. canis	Hd48	GQ395381
E. canis	NA	M73221

E. canis	NA	M73226
E. canis	Hd22	GQ395378
E. canis	Hd38-1	GQ395380
E. canis	ECAN_Bkk_07	EU263991
E. canis	NA	AF162860
E. canis	Jake	CP000107
E. canis	YZ-1	CP025749
E. chaffeensis	X1	KX505292
E. chaffeensis	Arkansas	NR_074500
E. chaffeensis	NA	AF147752
E. chaffeensis	NA	U23503
E. chaffeensis	Arkansas	NR_037059
E. chaffeensis	NA	AF000721
E. chaffeensis	**Arkansas**	**AF416764**
E. chaffeensis	NA	U60476
E. chaffeensis	NA	U86665
E. chaffeensis	NA	U86664
E. chaffeensis	NA	M73222
E. chaffeensis	West Paces	CP007480
E. chaffeensis	Wakulla	CP007479
E. chaffeensis	Saint Vincent	CP007478
E. chaffeensis	Osceola	CP007477
E. chaffeensis	Liberty	CP007476
E. chaffeensis	Jax	CP007475
E. chaffeensis	Heartland	CP007473
E. chaffeensis	Arkansas	CP000236
E. ewingii	Stillwater	NR_044747
E. ewingii	**Stillwater**	**M73227**
E. ewingii	95E9-TS	U96436
E. ewingii	95E7-Mk	U96435
E. muris	AS145	NR_025962
E. muris	AS145	NR_121714
E. muris	T-388	KU315171
E. muris	Est1709	KU535865
E. muris	WI22	HQ660491
E. muris	Ip16	AY587608
E. muris	NA	AB013009
E. muris	NA	AB196302
E. muris	Kh-1550	GU358692
E. muris	NA	AB013008
E. muris	Nov-Ip205	GU358691
E. muris	m12	AB275137
E. muris	NA	U15527
E. muris	**AS145**	**CP006917**
E. ruminantium	Welgevonden	NR_074513
E. ruminantium	AaFT299	KJ942239
E. ruminantium	AaFT77	KJ942221
E. ruminantium	Aa2FT306	KJ942213
E. ruminantium	SBF4	KF786044
E. ruminantium	SBF1	KF786042
E. ruminantium	Welgevonden	NR_074155
E. ruminantium	MB9_04	DQ640395
E. ruminantium	MB9_03	DQ640394
E. ruminantium	Hmr4	DQ640390
E. ruminantium	Sheep	DQ640389
E. ruminantium	Umbanein	DQ647616

E. ruminantium	c-4	DQ482922
E. ruminantium	c-3	DQ482921
E. ruminantium	c-2	DQ482920
E. ruminantium	c-1	DQ482919
E. ruminantium	c-3	DQ482918
E. ruminantium	c-2	DQ482917
E. ruminantium	c-1	DQ482916
E. ruminantium	c-1	DQ482915
E. ruminantium	NA	U03777
E. ruminantium	NA	X62432
E. ruminantium	**Welgevonden**	**CR925678**
E. ruminantium	Gardel	CR925677
E. ruminantium	Welgevonden	CR767821
E. ruminantium	Vista	AF318022
E. ruminantium	Henrique	AF318021
E. ruminantium	ribosomal	AF069758
E. ruminantium	rRNA	U03776
E. ruminantium	NA	X61659
E. ruminantium	S22	HQ908081
E. ruminantium	Kiswani	DQ647615
A. phagocytophilum	6952	KY486261
A. phagocytophilum	6097	KY486259
A. phagocytophilum	6403	KY486258
A. phagocytophilum	6403	KY462831
A. phagocytophilum	CPLA-HS	MG050134
A. phagocytophilum	DKRH-HS	MG050133
A. phagocytophilum	ItalyIRH004711	KY319198
A. phagocytophilum	Italy149-12B	KY319197
A. phagocytophilum	ItalyIRH012411	KY319196
A. phagocytophilum	ItalyIRH01221	KY319195
A. phagocytophilum	ItalyIRH004811	KY319194
A. phagocytophilum	ItalyH3th-28	KY319193
A. phagocytophilum	JXARSA-32	KU585968
A. phagocytophilum	JXARSA-2	KU585967
A. phagocytophilum	JXARSA-15	KU585966
A. phagocytophilum	HunChun17	KX279357
A. phagocytophilum	RB3	KY458571
A. phagocytophilum	RS10556	KY458570
A. phagocytophilum	Y_G14 16S	KU705164
A. phagocytophilum	Y_C35 16S	KU705163
A. phagocytophilum	Y_C34 16S	KU705162
A. phagocytophilum	Y_A04 16S	KU705161
A. phagocytophilum	I_BwReh33	KU705160
A. phagocytophilum	X_BwReh32	KU705159
A. phagocytophilum	16S-25	KU705130
A. phagocytophilum	W_BwRo6 1	KU705129
A. phagocytophilum	S_BwRo5 1	KU705128
A. phagocytophilum	W_BwRo4 1	KU705127
A. phagocytophilum	16S-30	KU705126
A. phagocytophilum	W_BwRo2 1	KU705125
A. phagocytophilum	Norway variant2, co	CP015376
A. phagocytophilum	ApGDrom1	KF002508
A. phagocytophilum	AAIK4	KR611719
A. phagocytophilum	AAIK3	KR611718
A. phagocytophilum	AAIK2	KR611717
A. phagocytophilum	AAIK1	KR611716

A. phagocytophilum	14DRS	KR092129
A. phagocytophilum	tick-EU329	KM215231
A. phagocytophilum	tick-EU322	KM215230
A. phagocytophilum	d-3216	KM215224
A. phagocytophilum	Aa2FT11	KJ942185
A. phagocytophilum	Aa2FT06	KJ942183
A. phagocytophilum	gw1	KF805344
A. phagocytophilum	Dog2	CP006618
A. phagocytophilum	JMe	CP006617
A. phagocytophilum	HZ2	CP006616
A. phagocytophilum	sheep19	KF293698
A. phagocytophilum	sheep28	KF293697
A. phagocytophilum	sheep29	KF293676
A. phagocytophilum	sheep54	KF293675
A. phagocytophilum	sheep52	KF293673
A. phagocytophilum	sheep50	KF293671
A. phagocytophilum	sheep49	KF293670
A. phagocytophilum	sheep48	KF293669
A. phagocytophilum	Rus30-10	HQ629911
A. phagocytophilum	Bel6-22-07	HQ629915
A. phagocytophilum	BelBmi37	HQ629914
A. phagocytophilum	Rus29-12	HQ629912
A. phagocytophilum	HB-SZ-HGA-S02	HQ171975
A. phagocytophilum	ZJ01/2008	HM439430
A. phagocytophilum	Sv-Ip854	HM366579
A. phagocytophilum	h997	HM138366
A. phagocytophilum	KWDAP5	GU556625
A. phagocytophilum	HLAP327	GU064899
A. phagocytophilum	KWDAP2	GU556622
A. phagocytophilum	KWDAP1	GU556621
A. phagocytophilum	sheep_5010_EM	GU236611
A. phagocytophilum	GC19	GU111744
A. phagocytophilum	roe_deer_137_05	GU236574
A. phagocytophilum	roe_deer_16	GU236554
A. phagocytophilum	YN-XD-HGA-S131	GQ500084
A. phagocytophilum	YN-XD-HGA-S103	GQ500080
A. phagocytophilum	YN-XD-HGA-S102	GQ500079
A. phagocytophilum	ZJ-TT-HGA-O37	GQ500060
A. phagocytophilum	ZJ-TT-HGA-O35	GQ500058
A. phagocytophilum	ZJ-TT-HGA-O23	GQ500047
A. phagocytophilum	BJ-MY-HGA-S13	GQ499986
A. phagocytophilum	BJ-MY-HGA-S2	GQ499977
A. phagocytophilum	BJ-MY-HGA-S1	GQ499956
A. phagocytophilum	AH-MG-HGA-S36	GQ499928
A. phagocytophilum	China-C-Aa	GQ412337
A. phagocytophilum	PoTiA2dt	EU098007
A. phagocytophilum	PoTiA1dt	EU098006
A. phagocytophilum	PoAnA1dt	EF693890
A. phagocytophilum	EHR02	EF217398
A. phagocytophilum	EHR1	EF217397
A. phagocytophilum	AH-HGA-9	EF473210
A. phagocytophilum	**HZ**	**CP000235**
A. phagocytophilum	M20	DQ361024
A. phagocytophilum	DBMGH	AY886761
A. phagocytophilum	474	AF481852
A. phagocytophilum	470	AF481850

A. phagocytophilum	AP-CBHL	AF470699
A. phagocytophilum	16S	AF384214
A. phagocytophilum	16S	AF384212
A. phagocytophilum	Jilin-1	DQ342324
A. phagocytophilum	BV-1	AY082656
A. phagocytophilum	TXCTR3	DQ088128
A. phagocytophilum	WA-variant	AY741095
A. phagocytophilum	h-1148	KM215222
A. platys	**Okinawa**	**AF536828**
A. platys	D35	KX792089
A. platys	4	KT982643
A. platys	QQQ	KX447505
A. platys	RRR	KX447502
A. platys	Rodo-p1-66	JX976181
A. platys	A.pl.#87	JQ396431
A. platys	Gigio	EU439943
A. platys	NA	EF139459
A. platys	YY36	MF289478
A. platys	YY33	MF289477
A. platys	b3-7	KY594914
A. platys	dog-72	LC269822
A. platys	dog-99	LC269821
A. platys	dog-166	LC269820
A. platys	WHBMXZ-126	KX987336
A. platys	ZJARSA-8	KU586183
A. platys	WHARSA-7	KU586175
A. platys	WHANSL-27-1	KU586172
A. platys	WHANSA-24-2	KU586168
A. platys	WHAEAP-26	KU586165
A. platys	WHAEAL-17-2	KU586163
A. platys	JXARSA-29	KU586161
A. platys	JXANSA-19	KU586159
A. platys	WHANSE-2	KU586124
A. platys	WHARSP-17	KU586058
A. platys	WHARSL-30	KU586051
A. platys	WHARSA-47-1	KU586031
A. platys	WHARSA-14	KU586028
A. platys	WHANSA-8	KU586006
A. platys	WHANSA-7-4	KU586001
A. platys	WHANSA-6-3	KU585997
A. platys	WHANSA-45-1	KU585989
A. platys	WHANSA-40	KU585988
A. platys	M48A	KX180946
A. platys	P30	KX180944
A. platys	NA	KY114935
A. platys	3ax1	KJ659045
A. platys	2ax1	KJ659044
A. platys	NA	AY530806
A. platys	Okinawa	AY077619
A. platys	NA	AF303467
A. platys	NA	AF399917
A. platys	NA	AF287153
A. platys	NA	AF286699
A. platys	NA	AF156784
A. platys	NA	M82801
A. marginale	BAGHS-B47	MG018436

A. marginale	Wayanad 17	MG728098
A. marginale	TVM 3	MG709131
A. marginale	Thrissur 8	MG709056
A. marginale	Thrissur 6	MG709054
A. marginale	Thrissur 5	MG709053
A. marginale	WHANSA-6-2	KU586171
A. marginale	WHAEAP-33	KU586166
A. marginale	JXANSA-2	KU586160
A. marginale	WHANSE-2-1	KU586125
A. marginale	ZJCUTA-30	KU586074
A. marginale	ZJCUTA-2	KU586073
A. marginale	ZJCUTA-10	KU586072
A. marginale	WHCUTA-25	KU586066
A. marginale	WHARSP-30-2	KU586062
A. marginale	WHARSP-19	KU586059
A. marginale	WHARSL-38-1	KU586056
A. marginale	WHARSA-47-2	KU586032
A. marginale	WHANSL-8-1	KU586022
A. marginale	WHANSL-27-2	KU586017
A. marginale	WHANSL-11-1	KU586012
A. marginale	WHANSA-92	KU586011
A. marginale	WHANSA-8-2	KU586005
A. marginale	WHANSA-60-2	KU585998
A. marginale	WHANSA-53-2	KU585995
A. marginale	WHANSA-48	KU585992
A. marginale	WHANSA-45-2	KU585990
A. marginale	JXANSA-8	KU585964
A. marginale	JXANSA-34	KU585963
A. marginale	JXANSA-24	KU585960
A. marginale	Uganda MT27	KU686794
A. marginale	Uganda MT28	KU686793
A. marginale	Uganda MT34	KU686792
A. marginale	Uganda MT30	KU686791
A. marginale	Uganda MT29	KU686790
A. marginale	Uganda MT31	KU686789
A. marginale	Uganda MT38	KU686788
A. marginale	Uganda KT7	KU686787
A. marginale	Uganda MT32	KU686786
A. marginale	Uganda MT37	KU686782
A. marginale	Uganda MT35	KU686781
A. marginale	Uganda MT33	KU686780
A. marginale	Uganda KT9	KU686779
A. marginale	Uganda MT42	KU686778
A. marginale	Uganda KT10	KU686777
A. marginale	Uganda KT6	KU686776
A. marginale	Uganda MT39	KU686775
A. marginale	Uganda MT36	KU686774
A. marginale	AM-SAR2011	KP877314
A. marginale	**Zaria**	**KJ095114**
A. marginale	NA	AB916498
A. marginale	NA	LC007100
A. marginale	NA	AB916499
A. marginale	Sivas SS101	KJ183086
A. marginale	Sivas SS84	KJ183083
A. marginale	NA	CP006847
A. marginale	NA	CP006846

A. marginale	C6A	JQ839012
A. marginale	C7D	JQ839011
A. marginale	5C	JQ839009
A. marginale	4C	JQ839008
A. marginale	ZJ02/2009	HM439433
A. marginale	K1	GU129918
A. marginale	NA	FJ155998
A. marginale	NA	CP001079
A. marginale	Ishigaki-2007	FJ226454
A. marginale	TG26	DQ000617
A. marginale	IG42	DQ000616
A. marginale	GP4	DQ000615
A. marginale	BS19	DQ000614
A. marginale	BS16	DQ000613
A. marginale	NA	AY048816
A. marginale	Hongan buffalo	DQ341369
A. marginale	16S	AJ633048
A. marginale	Zimbabwe	AF414878
A. marginale	Uruguay	AF414877
A. marginale	non-tailed	AF414875
A. marginale	F12	AF414874
A. marginale	Veld	AF414873
A. marginale	Eland	AF414872
A. marginale	South Africa	AF414871
A. marginale	NA	AF311303
A. marginale	South Idaho	AF309868
A. marginale	Virginia	AF309866
A. marginale	NA	CP000030
A. marginale	58	M60313
A. marginale	WHARSL-28	KU586048
A. marginale	ZJCUTA-6	KU586076
A. marginale	ZJCUTA-5	KU586075
A. marginale	WHARSA-30	KU586030
A. marginale	WHANSL-41	KU586021
A. marginale	WHCUTA-8	KU586067
A. marginale	WHANSL-24-1	KU586015
A. marginale	WHAEAL-17-1	KU585973
A. marginale	WHANSA-36	KU585984
A. marginale	CPY31	KM009068
A. marginale	AMSP4-MDK2	KX989511
A. marginale	AMSP4-MDK1	KX989510
A. marginale	HiP_5AM	KY305599
A. marginale	KNP_582AM	KY305598
A. ovis	BAGHS-B47	MG018436
A. ovis	Wayanad 17	MG728098
A. ovis	TVM 3	MG709131
A. ovis	Thrissur 8	MG709056
A. ovis	Thrissur 6	MG709054
A. ovis	Thrissur 5	MG709053
A. ovis	WHANSA-6-2	KU586171
A. ovis	WHAEAP-33	KU586166
A. ovis	JXANSA-2	KU586160
A. ovis	WHANSE-2-1	KU586125
A. ovis	ZJCUTA-30	KU586074
A. ovis	ZJCUTA-2	KU586073
A. ovis	ZJCUTA-10	KU586072

A. ovis	WHCUTA-25	KU586066
A. ovis	WHARSP-30-2	KU586062
A. ovis	WHARSP-19	KU586059
A. ovis	WHARSL-38-1	KU586056
A. ovis	WHARSA-47-2	KU586032
A. ovis	WHANSL-8-1	KU586022
A. ovis	WHANSL-27-2	KU586017
A. ovis	WHANSL-11-1	KU586012
A. ovis	WHANSA-92	KU586011
A. ovis	WHANSA-8-2	KU586005
A. ovis	WHANSA-60-2	KU585998
A. ovis	WHANSA-53-2	KU585995
A. ovis	WHANSA-48	KU585992
A. ovis	WHANSA-45-2	KU585990
A. ovis	JXANSA-8	KU585964
A. ovis	JXANSA-34	KU585963
A. ovis	JXANSA-24	KU585960
A. ovis	Uganda MT27	KU686794
A. ovis	Uganda MT28	KU686793
A. ovis	Uganda MT34	KU686792
A. ovis	Uganda MT30	KU686791
A. ovis	Uganda MT29	KU686790
A. ovis	Uganda MT31	KU686789
A. ovis	Uganda MT38	KU686788
A. ovis	Uganda KT7	KU686787
A. ovis	Uganda MT32	KU686786
A. ovis	Uganda MT37	KU686782
A. ovis	Uganda MT35	KU686781
A. ovis	Uganda MT33	KU686780
A. ovis	Uganda KT9	KU686779
A. ovis	Uganda MT42	KU686778
A. ovis	Uganda KT10	KU686777
A. ovis	Uganda KT6	KU686776
A. ovis	Uganda MT39	KU686775
A. ovis	Uganda MT36	KU686774
A. ovis	AM-SAR2011	KP877314
A. ovis	Zaria	KJ095114
A. ovis	NA	AB916498
A. ovis	NA	LC007100
A. ovis	NA	AB916499
A. ovis	Sivas SS101	KJ183086
A. ovis	Sivas SS84	KJ183083
A. ovis	NA	CP006847
A. ovis	NA	CP006846
A. ovis	C6A	JQ839012
A. ovis	C7D	JQ839011
A. ovis	5C	JQ839009
A. ovis	4C	JQ839008
A. ovis	ZJ02/2009	HM439433
A. ovis	**K1**	**GU129918**
A. ovis	NA	FJ155998
A. ovis	NA	CP001079
A. ovis	Ishigaki-2007	FJ226454
A. ovis	TG26	DQ000617
A. ovis	IG42	DQ000616
A. ovis	GP4	DQ000615

A. ovis	BS19	DQ000614
A. ovis	BS16	DQ000613
A. ovis	NA	AY048816
A. ovis	Hongan buffalo	DQ341369
A. ovis	16S	AJ633048
A. ovis	Zimbabwe	AF414878
A. ovis	Uruguay	AF414877
A. ovis	non-tailed	AF414875
A. ovis	F12	AF414874
A. ovis	Veld	AF414873
A. ovis	Eland	AF414872
A. ovis	South Africa	AF414871
A. ovis	NA	AF311303
A. ovis	South Idaho	AF309868
A. ovis	Virginia	AF309866
A. ovis	NA	CP000030
A. ovis	58	M60313
A. ovis	WHARSL-28	KU586048
A. ovis	ZJCUTA-6	KU586076
A. ovis	ZJCUTA-5	KU586075
A. ovis	WHARSA-30	KU586030
A. ovis	WHANSL-41	KU586021
A. ovis	WHCUTA-8	KU586067
A. ovis	WHANSL-24-1	KU586015
A. ovis	WHAEAL-17-1	KU585973
A. ovis	WHANSA-36	KU585984
A. ovis	CPY31	KM009068
A. ovis	AMSP4-MDK2	KX989511
A. ovis	AMSP4-MDK1	KX989510
A. ovis	HiP_5AM	KY305599
A. ovis	KNP_582AM	KY305598
A. centrale	NA	AF283007
A. centrale	16	EF520690
A. centrale	14	EF520689
A. centrale	8	EF520688
A. centrale	1	EF520687
A. centrale	CC	EF520686
A. centrale	**South Africa**	**AF414869**
A. centrale	Vaccine	AF414868
A. centrale	NA	AB211164
A. centrale	LP17	MF289482
A. centrale	LP10	MF289481
A. centrale	JJ5	MF289480
A. centrale	ZJ68	KP062966
A. centrale	ZJ64	KP062965
A. centrale	ZJ62	KP062964
A. centrale	Uganda KT5	KU686784
A. centrale	Uganda KT8	KU686783
A. centrale	C4B	JQ839010
A. centrale	HIP/A8/e	KC189842
A. centrale	HIP/A8/d	KC189841
A. centrale	HIP/A8/c	KC189840
A. centrale	NA	AB588977
A. centrale	HLAC222	GU064903
A. centrale	NA	AF318944
A. centrale	Israel	AF309869

A. bovis	Y258	KY425447
A. bovis	Y257	KY425445
A. bovis	Y201	KY425441
A. bovis	Y197	KY425439
A. bovis	Y196	KY425435
A. bovis	Y111	KY425433
A. bovis	Y102	KY425431
A. bovis	Y85	KY425429
A. bovis	Y83	KY425426
A. bovis	Y59	KY425423
A. bovis	Y11	KY425420
A. bovis	**Zhongxian**	**FJ169957**
A. bovis	SG176_HL	EU181143
A. bovis	SG175_HL	EU181142
A. bovis	Tottori-97	HM131218
A. bovis	Hiroshima-Z27	HM131217
A. bovis	ribosomal	AY144729
A. bovis	FL17	MF289479
A. bovis	Shandong JN2	KY242455
A. bovis	WHHLHP-119	KX987337
A. bovis	WHARSL-38-2	KU586176
A. bovis	WHARSA-40-2	KU586174
A. bovis	WHANSA-6-1	KU586170
A. bovis	WHANSA-24-1	KU586167
A. bovis	WHANSL-8	KU586023
A. bovis	20/China/2013	KP314253
A. bovis	19/China/2013	KP314252
A. bovis	18/China/2013	KP314251
A. bovis	17/China/2013	KP314250
A. bovis	15/China/2013	KP314249
A. bovis	14/China/2013	KP314248
A. bovis	13/China/2013	KP314247
A. bovis	12/China/2013	KP314246
A. bovis	11/China/2013	KP314245
A. bovis	10/China/2013	KP314244
A. bovis	9/China/2013	KP314243
A. bovis	8/China/2013	KP314242
A. bovis	6/China/2013	KP314240
A. bovis	4/China/2013	KP314239
A. bovis	1/China/2013	KP314236
A. bovis	ZJ98	KP062959
A. bovis	ZJ69	KP062958
A. bovis	ZJ66	KP062957
A. bovis	ZJ54	KP062956
A. bovis	ZJ46	KP062955
A. bovis	ZJ15	KP062954
A. bovis	ZJ12	KP062953
A. bovis	TYM19	LC012818
A. bovis	TYM14	LC012817
A. bovis	KUM7	LC012813
A. bovis	KUM5	LC012812
A. bovis	KOC4	LC012811
A. bovis	115	KM114613
A. bovis	85	KM114612
A. bovis	88	KM114611
A. bovis	sika35	LC060988

A. bovis	CMS-34_20111224	AB983439
A. bovis	CFT-27_20111223	AB983438
A. bovis	3ay	KJ659043
A. bovis	3ax	KJ659042
A. bovis	2ay	KJ659041
A. bovis	2ax	KJ659040
A. bovis	CFT-27-2010	AB723716
A. bovis	CFT-27-2009	AB723715
A. bovis	CFT-24-2010	AB723714
A. bovis	CFT-24-2009	AB723713
A. bovis	Am-vole57	JX092098
A. bovis	China-chipmunk25	JX092096
A. bovis	Kh-Hc215	JX092094
A. bovis	Am-Hc60	JX092092
A. bovis	Ab38B	AB588969
A. bovis	Ab59B	AB588965
A. bovis	raccoon513	GU937023
A. bovis	raccoon510	GU937022
A. bovis	raccoon501	GU937021
A. bovis	raccoon499	GU937020
A. bovis	raccoon493	GU937019
A. bovis	raccoon490	GU937018
A. bovis	raccoon464	GU937017
A. bovis	raccoon462	GU937016
A. bovis	raccoon458	GU937015
A. bovis	raccoon453	GU937014
A. bovis	raccoon439	GU937013
A. bovis	raccoon426	GU937012
A. bovis	raccoon109	GU937011
A. bovis	Yu12	JN558829
A. bovis	R7	JN558828
A. bovis	G55	JN558825
A. bovis	G49	JN558824
A. bovis	G21	JN558823
A. bovis	G1	JN558822
A. bovis	B7	JN558819
A. bovis	YX4	JN558817
A. bovis	G41	HQ913646
A. bovis	ES1090	HQ913645
A. bovis	ES1019	HQ913644
A. bovis	HLAB352	GU064902
A. bovis	HLAB187	GU064901
A. bovis	AB-KGHL	AF470698
A. bovis	NA	U03775
R. typhi	**Wilmington**	**L36221**
R. typhi	NA	M20499
R. typhi	Wilmington	U12463
R. rickettsii	NA	M21293
R. rickettsii	**R**	**L36217**
R. rickettsii	1995H02	DQ150694
R. rickettsii	1995H01	DQ150691
R. rickettsii	1994C02	DQ150688
R. rickettsii	1991C03	DQ150685
R. rickettsii	1989C01	DQ150682
R. rickettsii	NA	U11021

Supplementary Table S6. GenBank® accession codes for the *18S rRNA* gene sequences of *Babesia* and *Theileria* species. Sequences utilized for *in silico* analysis in Supplementary Fig. S1 are emphasized in bold.

***Babesia* Species**	**Isolate/Strain**	**GeneBank®**
B. canis vogeli	NA	HM590440
B. canis vogeli	NA	AY072925
B. canis vogeli	TWN2	HQ148664
B. canis vogeli	TWN1	HQ148663
B. canis vogeli	2B-158459	KY290979
B. canis vogeli	192704	KY290978
B. canis vogeli	105796	KY290977
B. canis vogeli	59239	KY290976
B. canis vogeli	dog 102	KT323936
B. canis vogeli	cat 100	KT323935
B. canis vogeli	dog 94	KT323934
B. canis vogeli	dog 86	KT323933
B. canis vogeli	cat 73	KT323932
B. canis vogeli	Belem Bv02	KT333456
B. canis vogeli	Dog#149	JX871891
B. canis vogeli	Dog#44	JX304683
B. canis vogeli	Dog#31	JX304682
B. canis vogeli	Dog#26	JX304681
B. canis vogeli	Dog#23	JX304680
B. canis vogeli	Dog#22	JX304679
B. canis vogeli	Dog#19	JX304677
B. canis vogeli	Guangxi	KJ939326
B. canis vogeli	SK-011	JX112785
B. canis vogeli	RO/FMVB/B/9	HQ662635
B. canis vogeli	NA	JF825145
B. canis vogeli	dog	AY150061
B. canis vogeli	NA	EU084681
B. canis vogeli	**Venezuela**	**DQ297390**
B. canis vogeli	Spain 1	DQ439545
B. canis vogeli	USA	AY371198
B. canis vogeli	Egypt	AY371197
B. canis vogeli	Brazil	AY371196
B. canis vogeli	Brazil	AY371195
B. canis vogeli	Brazil	AY371194
B. canis canis	Dog-1	KT008057
B. canis canis	BccTR2	KF499115
B. canis canis	2	EU622793
B. canis canis	1	EU622792
B. canis canis	NA	AY072926
B. canis canis	Bd6-2	AY962187
B. canis canis	Bd6-1	AY962186
B. canis canis	NA	AY649326
B. canis canis	**D5**	**AY527063**
B. canis canis	43 ldrf	DQ869308
B. canis canis	dog	DQ869307
B. canis canis	Dog#5	KC593879
B. canis canis	Dog#1	KC593878
B. canis canis	Dog#1	KC593877
B. canis canis	204A/13b	KP216422
B. canis canis	RO/FMVB/B/7	HQ662634
B. canis canis	BCC2	FJ209025

B. canis canis	BBC1	FJ209024
B. canis canis	NA	EU152128
B. canis canis	NA	AY321119
B. canis rossi	**Dog-76**	**DQ111764**
B. canis rossi	Dog-74	DQ111763
B. canis rossi	Dog-69	DQ111762
B. canis rossi	Dog-55	DQ111761
B. canis rossi	Dog-44	DQ111760
B. canis rossi	RLB1501/cl3	KY463434
B. canis rossi	RLB1535/cl1	KY463433
B. canis rossi	RLB1501/cl2	KY463432
B. canis rossi	RLB1501/cl1	KY463431
B. canis rossi	RLB1535/cl2	KY463430
B. canis rossi	RLB1535/cl3	KY463429
B. canis rossi	N4	AB935166
B. canis rossi	N3	AB935165
B. canis rossi	N2	AB935164
B. canis rossi	N1	AB935163
B. canis rossi	RLB67	JQ613105
B. canis rossi	RLB42	JQ613104
B. canis rossi	Dog#127, #135	AB303075
B. canis rossi	Dog#25, #354, #398	AB303074
B. canis rossi	Dog#23	AB303073
B. canis rossi	Dog#8	AB303072
B. canis rossi	Dog#2	AB303071
B. canis rossi	NA	KC453992
B. gibsoni	1-1665	KC461261
B. gibsoni	Yamaguchi dog-5672	AY077720
B. gibsoni	CMVL-06/2013/Chopin	KP901253
B. gibsoni	Assam 2	KF928958
B. gibsoni	Ludhiana 3	KF511956
B. gibsoni	Ludhiana 2	KF511955
B. gibsoni	Kolkata 2	KF171474
B. gibsoni	Kolkata 3	KF171473
B. gibsoni	**Siliguri**	**KF171471**
B. gibsoni	Dehradoon	KF171470
B. gibsoni	Kolkata	KJ142323
B. gibsoni	CMVL-05/2013/Kiki	KF878947
B. gibsoni	CMVL-01/2013/Mahesh	KF878943
B. gibsoni	218/Assam	KF112075
B. gibsoni	178/Assam	KF112074
B. gibsoni	TWN5	FJ769388
B. gibsoni	TWN4	FJ769387
B. gibsoni	TWN3	FJ769386
B. gibsoni	Bab/Asm-QM/11/Ind	KF606876
B. gibsoni	Bab/Asm-41/11/Ind	KF606867
B. gibsoni	Johor 314	KU500921
B. gibsoni	Pahang7	KU500920
B. gibsoni	Johor 282	KU500919
B. gibsoni	Sarawak 40	KU500918
B. gibsoni	Sarawak 18	KU500917
B. gibsoni	Selangor 53	KU500916
B. gibsoni	Sabah24	KU500915
B. gibsoni	WH123	KP666168
B. gibsoni	WH121	KP666167
B. gibsoni	WH120	KP666166

B. gibsoni	WH114	KP666165
B. gibsoni	WH110	KP666164
B. gibsoni	WH91	KP666163
B. gibsoni	WH87	KP666162
B. gibsoni	WH82	KP666161
B. gibsoni	WH71	KP666160
B. gibsoni	WH61	KP666159
B. gibsoni	WH58	KP666158
B. gibsoni	WH56	KP666157
B. gibsoni	WH54	KP666156
B. gibsoni	WH35	KP666155
B. gibsoni	BD37	LC008285
B. gibsoni	BD05	LC008284
B. gibsoni	BD02	LC006968
B. gibsoni	Punjab	KC954653
B. gibsoni	Kolkata-1	KC811803
B. gibsoni	Assam	KC811802
B. gibsoni	Bareilly-1	KC811801
B. gibsoni	TWN5	JQ710685
B. gibsoni	KOR07-20	EU430494
B. gibsoni	KOR06-104	EU430493
B. gibsoni	KOR06-102	EU430492
B. gibsoni	KOR0685	EU430491
B. gibsoni	KOR0675	EU430490
B. gibsoni	KOR0674	EU430489
B. gibsoni	KOR0656	EU430488
B. gibsoni	KOR0654-4	EU430487
B. gibsoni	KOR0644-2	EU430486
B. gibsoni	KOR0501-10	EU430481
B. gibsoni	NRCPD	AB478326
B. gibsoni	Aomori	AB118032
B. gibsoni	TWN1	EF587269
B. gibsoni	TWN2	EF587268
B. gibsoni	CMVL-01/2014	KY563118
B. gibsoni	CMVL Apr-13	KY524481
B. gibsoni	G135	LC169085
B. gibsoni	G112	LC169084
B. gibsoni	G111	LC169083
B. gibsoni	MB_P3	LC168621
B. gibsoni	MB_D2	LC168620
B. gibsoni	YGC17	LC012809
B. gibsoni	YGC15	LC012808
B. gibsoni	YGC14	LC012807
B. gibsoni	YGC11	LC012806
B. gibsoni	YGC10	LC012805
B. gibsoni	YGC8	LC012804
B. gibsoni	YGC7	LC012803
B. gibsoni	TKS20	LC012802
B. gibsoni	TKS5	LC012801
B. gibsoni	OSK7	LC012800
B. gibsoni	OSK3	LC012799
B. gibsoni	NGS6	LC012798
B. gibsoni	KUM4	LC012797
B. gibsoni	KUM3	LC012796
B. gibsoni	KUM2	LC012795
B. gibsoni	KGW18	LC012794

B. gibsoni	KGS4	LC012793
B. gibsoni	KGS1	LC012792
B. gibsoni	HNXY-2/2013	KJ715178
B. gibsoni	Nanjing1081	HG328237
B. gibsoni	Nanjing0010	HG328236
B. gibsoni	Nanjing0009	HG328235
B. gibsoni	SK-013	JX112784
B. gibsoni	Pig/J78	JX962780
B. gibsoni	Aydin	JN562745
B. gibsoni	11HS	EU084680
B. gibsoni	NA	EU084679
B. gibsoni	NA	EU084678
B. gibsoni	WM-1	EU084677
B. gibsoni	Okinawa dog-15	AY077718
B. bigemina	**Swiss_6**	**KM046917**
B. bigemina	JRHC-1	JX974332
B. bigemina	Umiam	KF112076
B. bigemina	Hs5	HQ688689
B. bigemina	Hs4	HQ688688
B. bigemina	Hs3	HQ688687
B. bigemina	Hs2	HQ688686
B. bigemina	Hs1	HQ688685
B. bigemina	Bab/Umi-H2/Ind	KF606866
B. bigemina	Bab/Umi-Hi/12/Ind	KF606865
B. bigemina	Bab/Umi-C2/12/Ind	KF606864
B. bigemina	Bab/Umi-T2/10/Ind	KF606863
B. bigemina	RG	JQ437264
B. bigemina	Bond	JQ437261
B. bigemina	ZJK17	KP710228
B. bigemina	TS103	KP710227
B. bigemina	CZ52	KP710226
B. bigemina	TJ09	KP710225
B. bigemina	SJZ26	KP710224
B. bigemina	MT26	KU206297
B. bigemina	MT25	KU206296
B. bigemina	KT4	KU206295
B. bigemina	MT24	KU206294
B. bigemina	MT23	KU206293
B. bigemina	MT22	KU206292
B. bigemina	MT21	KU206291
B. bigemina	TB5	KX115425
B. bigemina	Trkene3yumurta	KP745624
B. bigemina	Trkoz10	KP745623
B. bigemina	NA	JQ723014
B. bigemina	biLushi	JX495402
B. bigemina	PISJD34	JX104106
B. bigemina	563	HQ840960
B. bigemina	493	HQ840959
B. bigemina	B_bi19	EF458206
B. bigemina	B_bi18	EF458205
B. bigemina	B_bi17	EF458204
B. bigemina	B_bi16	EF458203
B. bigemina	B_bi14	EF458202
B. bigemina	B_bi12	EF458201
B. bigemina	B_bi11	EF458200
B. bigemina	B_bi10	EF458199

B. bigemina	B_bi09	EF458198
B. bigemina	B_bi08	EF458197
B. bigemina	B_bi07	EF458196
B. bigemina	B_bi06	EF458195
B. bigemina	B_bi05	EF458194
B. bigemina	B_bi04	EF458193
B. bigemina	B_bi03	EF458192
B. bigemina	B_bi02	EF458191
B. bigemina	B_bi01	EF458190
B. bigemina	BRC02	FJ426361
B. bigemina	Israel	EF612434
B. bigemina	Spain_1	DQ785311
B. microti	**US-Bm5**	**LC314658**
B. microti	US-Bm4	LC314657
B. microti	US-Bm3	LC314656
B. microti	US-Bm2	LC314655
B. microti	US-Bm1	LC314654
B. microti	IpSG13-18-1	LC127372
B. microti	IpSG13-16-3	LC127371
B. microti	IpSG13-10-2	LC127370
B. microti	IpSG13-1-2	LC127369
B. microti	Omsk-Tr17	KU955532
B. microti	Omsk-vole215_2015	KU955531
B. microti	Omsk-vole205_2015	KU955530
B. microti	Omsk-vole190_2015	KU955529
B. microti	Omsk-vole118_2013	KU955528
B. microti	Omsk-chipmunk2_2014	KU955527
B. microti	Omsk-vole130_2013	KU955526
B. microti	Omsk-vole92_2013	KU955525
B. microti	Omsk-vole57_2013	KU955524
B. microti	Omsk-vole45_2013	KU955523
B. microti	Omsk-vole24_2013	KU955522
B. microti	BmSSR218-3	LC005772
B. microti	BmSSR218-2	LC005771
B. microti	BmSSR218-1	LC005770
B. microti	BmSSR209-3	LC005769
B. microti	BmSSR209-1	LC005768
B. microti	BmSSR206-3	LC005767
B. microti	BmSSR206-2	LC005766
B. microti	BmSSR206-1	LC005765
B. microti	BmSSR190-5	LC005764
B. microti	BmSSR190-4	LC005763
B. microti	BmSSR190-3	LC005762
B. microti	BmSSR170-3	LC005761
B. microti	BmSSR170-2	LC005760
B. microti	BmSSR170-1	LC005759
B. microti	BmSSR168-3	LC005758
B. microti	BmSSR168-1	LC005757
B. microti	BmSSR167-9	LC005756
B. microti	BmSSR167-3	LC005755
B. microti	BmSSR167-1	LC005754
B. microti	BmSSR161-2	LC005753
B. microti	BmSSR159-1	LC005752
B. microti	TC-2012-C1	KF410827
B. microti	TC-2012-B99	KF410826
B. microti	TC-2012-B87	KF410825

B. microti	TC-2012-B1	KF410824
B. microti	Omsk-vole110	KC581934
B. microti	Yunnan-2	KC147723
B. microti	Yunnan-1	KC147722
B. microti	Ubl-104	AY943958
B. microti	Sbl-11	AY943957
B. microti	Gray	AY693840
B. microti	AF41002	AY918952
B. microti	AF2143	AY918951
B. microti	SN87-1	AY144702
B. microti	P8803	AY144701
B. microti	Rula	AY144700
B. microti	MT006	AY144699
B. microti	S837	AY144698
B. microti	Naushon	AY144697
B. microti	Nantucket	AY144696
B. microti	Pl2845Ips	AY144695
B. microti	Spooner	AY144694
B. microti	Russia	AY144693
B. microti	C5D182	AY144692
B. microti	VHSC1	AY144691
B. microti	GLS027Cg	AY144690
B. microti	400	AY144687
B. microti	NA	AB219802
B. microti	MM-1	EU168705
B. microti	Jena/Germany	EF413181
B. microti	NA	AB197940
B. microti	NA	AY094354
B. microti	Bm3	KX008036
B. microti	Bm2	KX008035
B. microti	Bm1	KX008034
B. microti	HLJ605	KU204798
B. microti	HLJ552	KU204797
B. microti	HLJ479	KU204796
B. microti	HLJ429	KU204795
B. microti	HLJ97	KU204794
B. microti	HLJ44	KU204793
B. microti	4IV	KT844560
B. microti	3IV	KT844559
B. microti	2IV	KT844558
B. microti	1IV	KT844557
B. microti	5BA	KT844556
B. microti	4BA	KT844555
B. microti	3BA	KT844554
B. microti	2BA	KT844553
B. microti	NA	KT867773
B. microti	NA	KT318132
B. microti	Irk-Ip332	KJ486556
B. microti	CNMM-2	AB736270
B. microti	CNMM-1	AB731747
B. microti	Yunnan-3	KC147724
B. microti	Rat/6	JX962781
B. microti	Fox/J28	JX962779
B. microti	hlj72	JQ993429
B. microti	449-L	JQ609304
B. microti	JM1	AB576641

B. microti	Nov-Ip307	GU057383
B. microti	NA	AY789075
B. microti	Kh-1026	GU057386
B. microti	Kh-Ip67	GU057384
B. microti	RI	XR_002459986
B. caballi	**B1_CABRBEQ179**	**EU642513**
B. caballi	A_CABEQ30	EU642512
B. caballi	B1_CABRBEQ164	EU888904
B. caballi	CABEQ51_B1	EU888901
B. caballi	CABEQ50_B1	EU888900
B. caballi	B2_CABRBEQ115	EU642514
B. caballi	NA	KJ549665
B. caballi	EqP-117	JX049130
B. caballi	NA	MF384422
B. caballi	Jaboticabal	KY952238
B. caballi	142	KY952236
B. caballi	157	KY952235
B. caballi	151	KY952234
B. caballi	143	KY952233
B. caballi	Bacab5	KX375825
B. caballi	Bacab1	KX375824
B. caballi	U244	AB734409
B. caballi	A134	AB734408
B. caballi	A135	AB734407
B. caballi	A139	AB734406
B. caballi	A195	AB734405
B. caballi	A161	AB734404
B. caballi	A188	AB734403
B. caballi	A142	AB734402
B. caballi	U237	AB734401
B. caballi	U243	AB734400
B. caballi	U244	AB734399
B. caballi	U232	AB734398
B. caballi	U231	AB734397
B. caballi	T23	AB734396
B. caballi	U240	AB734395
B. caballi	A214	AB734394
B. caballi	A158	AB734393
B. caballi	A123	AB734392
B. caballi	T103	AB734391
B. caballi	T90	AB734390
B. caballi	T64	AB734389
B. caballi	T48	AB734388
B. caballi	T16	AB734387
B. caballi	T4	AB734386
B. caballi	NA	FJ209026
B. caballi	NA	AY309955
B. caballi	EB1	AY534883
B. caballi	NA	Z15104
B. divergens	NA	AY144688
B. divergens	**Spanish_2**	**MG944238**
B. divergens	IpSG14-2-2	LC279018
B. divergens	IPSG13-13-1	AB975389
B. divergens	Trbrt35	KP745627
B. divergens	IpSG10	KC493555
B. divergens	C139	FJ944826

B. divergens	1802A	FJ944825
B. divergens	Bob2	FJ944824
B. divergens	CF2000	FJ944823
B. divergens	Rouen 87	FJ944822
B. divergens	B2	EU182595
B. divergens	B1	EU182594
B. divergens	HLJ216	KU377437
B. divergens	HLJ216	KU204799
B. divergens	R105	KM657258
B. divergens	Spanish	KF533077
B. divergens	Aug-43	AB861507
B. divergens	Aug-40	AB861506
B. divergens	18-Aug	AB861505
B. divergens	4-Aug	AB861504
B. divergens	Aug-38	AB857846
B. divergens	Jul-33	AB857845
B. divergens	25-Aug	KC465974
B. divergens	12-Jul	KC465977
B. divergens	Aug-41	KC465976
B. divergens	20-Jul	KC465975
B. divergens	Aug-51	KC465973
B. divergens	RD54	JQ929916
B. divergens	CVD7	GQ304525
B. divergens	CVD2	GQ304524
B. divergens	Nov-Ip316	GU057385
B. divergens	NA	AY789076
B. divergens	BAB105	AY046576
B. divergens	B_di09	EF458228
B. divergens	B_di10	EF458229
B. divergens	B_di08	EF458227
B. divergens	B_di07	EF458226
B. divergens	B_di06	EF458225
B. divergens	B_di05	EF458224
B. divergens	B_di04	EF458223
B. divergens	B_di03	EF458222
B. divergens	B_di02	EF458221
B. divergens	B_di01	EF458220
B. divergens	B_di11	EF458219
B. divergens	NA	AY572456
B. divergens	NA	AJ439713
B. divergens	NA	U16370
B. divergens	NA	Z48751
B. bovis	**BRC01**	**FJ426364**
B. bovis	Bareilly	KF928959
B. bovis	BBOV2	L19077
B. bovis	BBOV3	L19078
B. bovis	USDA	HQ264112
B. bovis	USDA	HQ264111
B. bovis	H8	HQ264110
B. bovis	NR8	HQ264106
B. bovis	NR8	HQ264105
B. bovis	USDA	GU906886
B. bovis	Merida	GU906885
B. bovis	Merida	GU906884
B. bovis	Merida	GU906883
B. bovis	H81	JQ437262

B. bovis	8284	JQ437260
B. bovis	ZJK15	KP710223
B. bovis	TS35	KP710222
B. bovis	Trbrt36	KP745628
B. bovis	NA	JQ723013
B. bovis	boLushi	JX495403
B. bovis	cow	AY150059
B. bovis	B_bo18	EF458218
B. bovis	B_bo17	EF458217
B. bovis	B_bo16	EF458216
B. bovis	B_bo10	EF458215
B. bovis	B_bo09	EF458214
B. bovis	B_bo08	EF458213
B. bovis	B_bo07	EF458212
B. bovis	B_bo05	EF458211
B. bovis	B_bo03	EF458210
B. bovis	B_bo01	EF458209
B. bovis	B_bo19	EF458208
B. conradae	**NA**	**AF158702**
B. vulpes	**1061L**	**KJ871352**
B. vulpes	910L	KJ871351
B. vulpes	03/00349	KT223483
B. duncani	**BAB1615**	**HQ289870**
B. duncani	Bdu	KX008042
B. duncani	Bdi	KX008037
B. duncani	BAB2	HQ285838
Theileria* (*Babesia*) *eqeui	**RJ20**	**KJ573374**
Theileria (*Babesia*) *eqeui*	RJ19	KJ573373
Theileria (*Babesia*) *eqeui*	RJ15	KJ573372
Theileria (*Babesia*) *eqeui*	RJ4	KJ573371
Theileria (*Babesia*) *eqeui*	RJ2	KJ573370
Theileria (*Babesia*) *eqeui*	B_LFEQ164	EU642511
Theileria (*Babesia*) *eqeui*	B_LFEQ47	EU642510
Theileria (*Babesia*) *eqeui*	B_RBEQ105	EU642509
Theileria (*Babesia*) *eqeui*	A1_RBEQ178	EU642508
Theileria (*Babesia*) *eqeui*	A2_RBEQ101	EU642507
Theileria (*Babesia*) *eqeui*	LFEQ23_A	EU888906
Theileria (*Babesia*) *eqeui*	LFEQ178_C	EU888905
Theileria (*Babesia*) *eqeui*	LFEQ189_C	EU888903
Theileria (*Babesia*) *eqeui*	RBEQ63_A	EU888902
Theileria (*Babesia*) *eqeui*	TE/18s/GUJ	KP995259
Theileria (*Babesia*) *eqeui*	Te0022_CA	JX177673
Theileria (*Babesia*) *eqeui*	Te0001_FL	JX177672
Theileria (*Babesia*) *eqeui*	Te0002_TX	JX177671
Theileria (*Babesia*) *eqeui*	Te0004_TX	JX177670
Theileria (*Babesia*) *eqeui*	Ho233	JQ390047
Theileria (*Babesia*) *eqeui*	NA	KF559357
Theileria (*Babesia*) *eqeui*	203-7	JQ657703
Theileria (*Babesia*) *eqeui*	KNU	HM229408
Theileria (*Babesia*) *eqeui*	KNU	HM229407
Theileria (*Babesia*) *eqeui*	ET1	AY534882
Theileria (*Babesia*) *eqeui*	H16	KY111762
Theileria (*Babesia*) *eqeui*	H12	KY111761
Theileria (*Babesia*) *eqeui*	H01	KY111760
Theileria (*Babesia*) *eqeui*	RJ18	KX722525
Theileria (*Babesia*) *eqeui*	RJ3	KX722524

Theileria (*Babesia*) *eqeui*	RJ17	KX722523
Theileria (*Babesia*) *eqeui*	RJ16	KX722522
Theileria (*Babesia*) *eqeui*	RJ14	KX722521
Theileria (*Babesia*) *eqeui*	RJ13	KX722520
Theileria (*Babesia*) *eqeui*	RJ12	KX722519
Theileria (*Babesia*) *eqeui*	RJ11	KX722518
Theileria (*Babesia*) *eqeui*	RJ10	KX722517
Theileria (*Babesia*) *eqeui*	RJ9	KX722516
Theileria (*Babesia*) *eqeui*	RJ8	KX722515
Theileria (*Babesia*) *eqeui*	RJ7	KX722514
Theileria (*Babesia*) *eqeui*	RJ6	KX722513
Theileria (*Babesia*) *eqeui*	RJ5	KX722512
Theileria (*Babesia*) *eqeui*	RJ1	KX722511
Theileria (*Babesia*) *eqeui*	A11N	KX227641
Theileria (*Babesia*) *eqeui*	156N	KX227640
Theileria (*Babesia*) *eqeui*	347C	KX227639
Theileria (*Babesia*) *eqeui*	B1	KX227638
Theileria (*Babesia*) *eqeui*	3011C	KX227637
Theileria (*Babesia*) *eqeui*	U4S	KX227636
Theileria (*Babesia*) *eqeui*	U2S	KX227635
Theileria (*Babesia*) *eqeui*	T1N	KX227634
Theileria (*Babesia*) *eqeui*	PA20	KX227633
Theileria (*Babesia*) *eqeui*	PA13	KX227632
Theileria (*Babesia*) *eqeui*	PA46	KX227631
Theileria (*Babesia*) *eqeui*	O5S	KX227630
Theileria (*Babesia*) *eqeui*	O2S	KX227629
Theileria (*Babesia*) *eqeui*	G6	KX227628
Theileria (*Babesia*) *eqeui*	F14C	KX227627
Theileria (*Babesia*) *eqeui*	E37C	KX227626
Theileria (*Babesia*) *eqeui*	5512N	KX227625
Theileria (*Babesia*) *eqeui*	177S	KX227624
Theileria (*Babesia*) *eqeui*	101J	KX227623
Theileria (*Babesia*) *eqeui*	PA21	KX227622
Theileria (*Babesia*) *eqeui*	20J	KX227621
Theileria (*Babesia*) *eqeui*	9C	KX227620
Theileria (*Babesia*) *eqeui*	459N	KX227619
Theileria (*Babesia*) *eqeui*	Ho-79	KU672386
Theileria (*Babesia*) *eqeui*	Swiss-5	KM046922
Theileria (*Babesia*) *eqeui*	Swiss-4	KM046921
Theileria (*Babesia*) *eqeui*	Swiss-3	KM046920
Theileria (*Babesia*) *eqeui*	Swiss-2	KM046919
Theileria (*Babesia*) *eqeui*	Swiss-1	KM046918
Theileria (*Babesia*) *eqeui*	JJ42	KM819520
Theileria (*Babesia*) *eqeui*	El Obied46	AB515315
Theileria (*Babesia*) *eqeui*	Kosti5	AB515314
Theileria (*Babesia*) *eqeui*	Omdurman2	AB515313
Theileria (*Babesia*) *eqeui*	Khartoum North15	AB515312
Theileria (*Babesia*) *eqeui*	Atbara13	AB515311
Theileria (*Babesia*) *eqeui*	Atbara8	AB515310
Theileria (*Babesia*) *eqeui*	Atbara5	AB515309
Theileria (*Babesia*) *eqeui*	Atbara2	AB515308
Theileria (*Babesia*) *eqeui*	Atbara1	AB515307
Theileria (*Babesia*) *eqeui*	T42	AB733379
Theileria (*Babesia*) *eqeui*	T10	AB733378
Theileria (*Babesia*) *eqeui*	U225	AB733377
Theileria (*Babesia*) *eqeui*	T95	AB733376

Theileria (*Babesia*) *eqeui*	A200	AB733375
Theileria (*Babesia*) *eqeui*	T8	AB733374
Theileria (*Babesia*) *eqeui*	A129	AB733373
Theileria (*Babesia*) *eqeui*	A128	AB733372
Theileria (*Babesia*) *eqeui*	dog	AY150064
Theileria (*Babesia*) *eqeui*	Spain-2	AY150063
Theileria (*Babesia*) *eqeui*	Spain-1	AY150062
Theileria (*Babesia*) *eqeui*	Spain-3	DQ287951
Theileria (*Babesia*) *eqeui*	NA	Z15105
Cytauxzoon felis	NA	L19080
Cytauxzoon felis	**309**	**KU306948**
Cytauxzoon felis	307	KU306947
Cytauxzoon felis	303	KU306946
Cytauxzoon felis	53aug	KU306945
Cytauxzoon felis	26-Aug	KU306944
Cytauxzoon felis	20-Aug	KU306943
Cytauxzoon felis	18-Aug	KU306942
Cytauxzoon felis	13-Aug	KU306941
Cytauxzoon felis	7-Oct	KU306940
Cytauxzoon felis	NA	AF399930
Cytauxzoon felis	NA	GU903911

Supplementary Table S7. GenBank® accession codes for the *bipA* gene sequences of *Borrelia* species. Sequences utilized for *in silico* analysis in Supplementary Fig. S1 are emphasized in bold.

***Borrelia* Species**	**Isolate/Strain**	**GeneBank®**
B. turicatae	**FCB-1**	**C845531**
B. turicatae	NA	GU270942
B. turicatae	91E135	HM008710
B. turicatae	95PE570	KC845528
B. turicatae	TCB-2	KC845530
B. turicatae	95PE1807	KC845527
B. turicatae	BTE5EL	CP015630
B. turicatae	**TCB-1**	**KC845529**
B. parkeri	**HR1**	**CP007036**
B. hermsii	SWA	GQ869822
B. hermsii	SIS	GQ869821
B. hermsii	RUM	GQ869820
B. hermsii	REN	GQ869819
B. hermsii	RAL	GQ869818
B. hermsii	OKA-3	GQ869817
B. hermsii	OKA-2	GQ869816
B. hermsii	OKA-1	GQ869815
B. hermsii	MTW-4	GQ869814
B. hermsii	MTW-3	GQ869813
B. hermsii	MTW-2	GQ869812
B. hermsii	MIL	GQ869811
B. hermsii	MCN	GQ869810
B. hermsii	MAT	GQ869809
B. hermsii	Man	GQ869808
B. hermsii	LAK-5	GQ869807
B. hermsii	LAK-4	GQ869806
B. hermsii	LAK-3	GQ869805
B. hermsii	LAK-2	GQ869804
B. hermsii	LAK-1	GQ869803
B. hermsii	HS1	GQ869802

B. hermsii	HAN	GQ869801
B. hermsii	HAL	GQ869800
B. hermsii	GMC	GQ869799
B. hermsii	GAR	GQ869798
B. hermsii	FRS	GQ869797
B. hermsii	FRO	GQ869796
B. hermsii	FRE	GQ869795
B. hermsii	EST	GQ869794
B. hermsii	CON	GQ869792
B. hermsii	CMC	GQ869791
B. hermsii	CAR	GQ869790
B. hermsii	BYM	GQ869789
B. hermsii	BRO	GQ869788
B. hermsii	BAK	GQ869787
B. hermsii	ALL	GQ869786
B. hermsii	**YOR**	**GQ869824**
B. hermsii	**DAH**	**GQ869793**
B. hermsii	DAH	FJ446703
B. hermsii	WAD	GQ869823

Supplementary Table S8. GenBank® accession codes for the *flaB* gene sequences of *Borrelia* species. Sequences utilized for *in silico* analysis in Supplementary Fig. S1 are emphasized in bold.

***Borrelia* Species**	**Isolate/Strain**	**GeneBank®**
B. turicatae	FCB	AY934630
B. turicatae	**TCB-2**	**AY934629**
B. turicatae	TCB-1	AY934628
B. turicatae	PE1-926	AY934627
B. turicatae	99PE-1807	AY934626
B. turicatae	95PE-570	AY934625
B. turicatae	91E135	AY934624
B. turicatae	91E135	AY604979
B. turicatae	91E135	NC_008710
B. parkeri	CA221	AY934623
B. parkeri	CA220	AY934622
B. parkeri	CA219	AY934621
B. parkeri	CA218	AY934620
B. parkeri	CA216	AY934619
B. parkeri	RML	AY604980
B. parkeri	HR1	CP007022
B. parkeri	**SLO**	**NZ_CP005851**
B. miyamotoi	HoHe	KT932823
B. miyamotoi	ARH554	KT452933
B. miyamotoi	ARH624	KT452932
B. miyamotoi	ARH615	KT452930
B. miyamotoi	ARH657	KT452931
B. miyamotoi	CT13-2396	CP017126
B. miyamotoi	ARH656	KT452934
B. miyamotoi	Izh-16	CP024351
B. miyamotoi	Izh-4	CP024390
B. miyamotoi	Yekat-6	CP024316
B. miyamotoi	Izh-5	CP024205
B. miyamotoi	Izh-14	CP024371
B. miyamotoi	Yekat-1	CP024333
B. miyamotoi	**FR64b**	**CP004217**
B. miyamotoi	CA17-2241	CP021872

B. miyamotoi	13T392	KU749379
B. miyamotoi	15H532	KU749378
B. coriaceae	NA	DQ320140
B. coriaceae	**Co53**	**NZ_CP005745**
B. anserina	5AY_H01_8-125ans_E_G03	KY438930
B. anserina	Es	CP013704
B. anserina	PL	DQ849626
B. anserina	**BA2**	**CP005829**
B. crocidurae	DOS-27	JX292911
B. crocidurae	DOU-1b	JX292912
B. crocidurae	DOU-686	JX292913
B. crocidurae	DOU-690	JX292914
B. crocidurae	DOU	JX292915
B. crocidurae	DOS-2	JX292916
B. crocidurae	DOS-3	JX292917
B. crocidurae	DOS-6	JX292919
B. crocidurae	DOS-5	JX292918
B. crocidurae	DOS-7	JX292920
B. crocidurae	DOS-13	JX292921
B. crocidurae	DOS-16	JX292922
B. crocidurae	Achema	GU357619
B. crocidurae	**DOS-56**	**JX292925**
B. crocidurae	KOS-46	JX292924
B. crocidurae	KOS-39	JX292923
B. crocidurae	Achema	CP003426
B. recurrentis	**A1**	**DQ346814**
B. recurrentis	107	AY604982
B. recurrentis	A2	DQ346815
B. recurrentis	A3	DQ346816
B. recurrentis	A4	DQ346817
B. recurrentis	A6	DQ346819
B. recurrentis	A5	DQ346818
B. recurrentis	A18	DQ346831
B. recurrentis	A17	DQ346830
B. recurrentis	A16	DQ346829
B. recurrentis	A15	DQ346828
B. recurrentis	A14	DQ346827
B. recurrentis	A13	DQ346826
B. recurrentis	A12	DQ346825
B. recurrentis	A11	DQ346824
B. recurrentis	A10	DQ346823
B. recurrentis	A9	DQ346822
B. recurrentis	A8	DQ346821
B. recurrentis	A7	DQ346820
B. recurrentis	A1	CP000993
B. burgdorferi	DG1-04	DQ016625
B. burgdorferi	GL56-07	HM345910
B. burgdorferi	D69-04	DQ016620
B. burgdorferi	T90-5-02	HM345911
B. burgdorferi	Bb_V2	MF150052
B. burgdorferi	Bb_V3	MF150053
B. burgdorferi	Bb_V6	MF150055
B. burgdorferi	Bb_V5	MF150054
B. burgdorferi	Bb_V7	MF150056
B. burgdorferi	Tr293	AB091813
B. burgdorferi	NA	AB189460

B. burgdorferi	CA8	GQ247741
B. burgdorferi	B331	CP017201
B. burgdorferi	Pabe	CP019916
B. burgdorferi	Pali	CP019844
B. burgdorferi	B31-5A1	CP009656
B. burgdorferi	CA382	CP005925
B. burgdorferi	N40	CP002228
B. burgdorferi	JD1	CP002312
B. burgdorferi	ZS7	CP001205
B. burgdorferi	**B31**	**AE000783**
B. afzelii	A1_Aag_Waw	KY626318
B. afzelii	A1_Afl_Waw	KY626319
B. afzelii	B1_Aag_Waw	KY626320
B. afzelii	B2_Aag_Waw	KY626321
B. afzelii	B3_Aag_Waw	KY626322
B. afzelii	B4_Aag_Waw	KY626323
B. afzelii	B5_Aag_Waw	KY626324
B. afzelii	B6_Afl_Waw	KY626325
B. afzelii	Ba_V1a	MF150047
B. afzelii	Ba_V1b	MF150048
B. afzelii	Ba_V2cc	MF150049
B. afzelii	Ba_v2ct	MF150050
B. afzelii	Ba_V2tc	MF150051
B. afzelii	NA	AB236667
B. afzelii	Tr38	AB091806
B. afzelii	Tr96	AB091808
B. afzelii	NA	AB189459
B. afzelii	DB19N7-04	GQ918147
B. afzelii	41-M-11	KF894063
B. afzelii	9W10-04	FJ874924
B. afzelii	Mr11	AB178334
B. afzelii	Mp6	AB178335
B. afzelii	29-MD-11	KF894069
B. afzelii	21-O-12	KF894067
B. afzelii	25-M-11	KF894068
B. afzelii	14-MD-12	KF894066
B. afzelii	9-S-11	KF894065
B. afzelii	80-M-11	KF894064
B. afzelii	CFC8E	AB178780
B. afzelii	Mp4	AB178779
B. afzelii	51-S-12	KF894070
B. afzelii	OS17-07	HM345909
B. afzelii	ST19-05	HM345908
B. afzelii	ZL109-07	HM345907
B. afzelii	**BO23**	**CP018262**
B. afzelii	K78	CP009058
B. afzelii	Tom3107	CP009212
B. afzelii	HLJ01	CP003882
B. afzelii	Pko	CP002933
B. afzelii	Pko	CP000395
B. garinii	D106-04	DQ016621
B. garinii	D7-04	DQ016622
B. garinii	DB1F7-04	DQ650331
B. garinii	DB18N6-04	DQ650333
B. garinii	T32-5-05	DQ650336
B. garinii	PB35-99	HM345897

B. garinii	DB74-01	HM345898
B. garinii	T40-10-02	HM345902
B. garinii	T53-9-02	HM345901
B. garinii	ZL148-07	HM345900
B. garinii	DB60-01	HM345899
B. garinii	RP54-05	HM345906
B. garinii	ST12-05	HM345905
B. garinii	T44-4-02	HM345904
B. garinii	T41-2-02	HM345903
B. garinii	Bg_vA	MF150057
B. garinii	Bg_vAa	MF150058
B. garinii	Bg_vC	MF150062
B. garinii	Bg_vBb	MF150061
B. garinii	Bg_vBa	MF150060
B. garinii	Bg_vB	MF150059
B. garinii	Bg_vDf	MF150069
B. garinii	Bg_vDe	MF150068
B. garinii	Bg_vDd	MF150067
B. garinii	Bg_vDc	MF150066
B. garinii	Bg_vDb	MF150065
B. garinii	Bg_vDa	MF150064
B. garinii	Bg_vCa	MF150063
B. garinii	Bg_vEa	MF150070
B. garinii	Bg_vEb	MF150071
B. garinii	Bg_vEc	MF150072
B. garinii	Bg_vEd	MF150073
B. garinii	Bg_vEf	MF150074
B. garinii	Tr77	AB091807
B. garinii	Tr309	AB091814
B. garinii	Mr8	AB178325
B. garinii	Mr3	AB178326
B. garinii	Mr10	AB178327
B. garinii	Nr267	AB178328
B. garinii	Mp5	AB178329
B. garinii	Mr12	AB178330
B. garinii	23-M-11	KF894053
B. garinii	39-M-11	KF894052
B. garinii	Mp7	AB178332
B. garinii	61-M-11	KF894058
B. garinii	56-M-11	KF894057
B. garinii	67-S-12	KF894056
B. garinii	66-S-12	KF894055
B. garinii	60-S-12	KF894054
B. garinii	77-M-11	KF894061
B. garinii	58-M-11	KF894060
B. garinii	55-M-11	KF894059
B. garinii	Nsk-10-06	EU979630
B. garinii	**SZ**	**CP007564**
B. hermsii GGI	LAK-4	DQ855533
B. hermsii GGI	ELD	DQ855531
B. hermsii GGI	HS1	EF583449
B. hermsii GGI	HS1	GU357620
B. hermsii GGI	WAD	AY597794
B. hermsii GGI	MAN	AY597793
B. hermsii GGI	ALL	AY597791
B. hermsii GGI	GAR	AY597790

B. hermsii GGI	FR0	AY597789
B. hermsii GGI	SIS	AY597788
B. hermsii GGI	RAL	AY597787
B. hermsii GGI	BAK	AY597786
B. hermsii GGI	BYM	AY597785
B. hermsii GGI	CAR	AY597784
B. hermsii GGI	SWA	AY597783
B. hermsii GGI	FRE	AY597782
B. hermsii GGI	C0N	AY597781
B. hermsii GGI	HAL	AY597780
B. hermsii GGI	BR0	AY597779
***B. hermsii* GGI**	**DAH**	**AY597777**
B. hermsii GGI	HS1	CP014349
B. hermsii GGI	MIT-24	KX171817
B. hermsii GGI	YBT-21	KX171812
B. hermsii GGI	YBT-12	KX171807
B. hermsii GGI	YBT-7	KX171805
B. hermsii GGI	YBS-1171	KX171804
B. hermsii GGI	WHT-8	KX171798
B. hermsii GGI	DM-31	KX171797
B. hermsii GGI	WHS-90	KX171796
B. hermsii GGI	LAK-6	KX171792
B. hermsii GGI	WAR	KC883464
B. hermsii GGI	CC1	JF737019
B. hermsii GGI	EST	AY597792
B. hermsii GGI	OWL	GQ175063
B. hermsii GGI	YB-Th-60	GQ175059
B. hermsii GGI	HCT-4	KJ995789
B. hermsii GGI	COR	KJ995774
B. hermsii GGI	DAH 2E7	CP014808
B. hermsii GGII	LAK-5	DQ855534
B. hermsii GGII	LAK-3	DQ855532
B. hermsii GGII	REN	AY597805
B. hermsii GGII	OKA-3	AY597804
B. hermsii GGII	OKA-2	AY597803
B. hermsii GGII	OKA-1	AY597802
B. hermsii GGII	HAN	AY597801
B. hermsii GGII	SIL	AY597800
B. hermsii GGII	LAK-2	AY597799
B. hermsii GGII	LAK-1	AY597798
B. hermsii GGII	RUM	AY597797
B. hermsii GGII	GMC	AY597796
B. hermsii GGII	CMC	AY597795
***B. hermsii* GGII**	**YOR**	**AY597806**
B. hermsii GGII	MAT	DQ855535
B. hermsii GGII	MIL	AY597778
B. hermsii GGII	YBS-60	KX171799
B. hermsii GGII	MIT-27	KX171816
B. hermsii GGII	MIT-26	KX171815
B. hermsii GGII	MIS-491	KX171814
B. hermsii GGII	MIS-1014	KX171813
B. hermsii GGII	YBT-20	KX171811
B. hermsii GGII	YBT-18	KX171810
B. hermsii GGII	YBT-17	KX171809
B. hermsii GGII	YBT-13	KX171808
B. hermsii GGII	YBT-10	KX171806

B. hermsii GGII	YBS-1143	KX171803
B. hermsii GGII	YBS-479	KX171802
B. hermsii GGII	YBS-266	KX171801
B. hermsii GGII	YBS-70	KX171800
B. hermsii GGII	WHS-88	KX171795
B. hermsii GGII	WHS-81	KX171794
B. hermsii GGII	WHS-40	KX171793
B. hermsii GGII	WCB-1	KC883463
B. hermsii GGII	LP0	EF595741
B. hermsii GGII	MTW-2	EU194843
B. hermsii GGII	MTW-1	EU194839
B. hermsii GGII	COT-7	KJ995784

Supplementary Table S9. GenBank® accession codes for the *msp2* gene sequences of *Anaplasma phagocytophilum*. Sequences utilized for *in silico* analysis in Supplementary Fig. S1 are emphasized in bold.

***Anaplasma* Species**	**Isolate/Strain**	**GeneBank®**
A. phagocytophilum	**HZ**	**CP000235**
A. phagocytophilum	AP-V1	AY780258
A. phagocytophilum	LL	AY568558
A. phagocytophilum	MRK	AY568557
A. phagocytophilum	NY-31	AY541007
A. phagocytophilum	NY-36	AY541006
A. phagocytophilum	NY-37	AY541005
A. phagocytophilum	OS	AY541004
A. phagocytophilum	Trustom	AY541003
A. phagocytophilum	AVK-HLPA1	AY541002
A. phagocytophilum	Gaillard	AY541001
A. phagocytophilum	MN-2	AY541000
A. phagocytophilum	MN-9	AY540999
A. phagocytophilum	S1025/09	JF893912
A. phagocytophilum	S2630/07	JF893911
A. phagocytophilum	S2614/07	JF893910
A. phagocytophilum	S1829/04	JF893909
A. phagocytophilum	S1741/07	JF893908
A. phagocytophilum	S1729/08	JF893907
A. phagocytophilum	S1523/07	JF893906
A. phagocytophilum	S1379/06	JF893905
A. phagocytophilum	S1220/08	JF893904
A. phagocytophilum	S1201/05	JF893903
A. phagocytophilum	S1085/09	JF893902
A. phagocytophilum	S1074/09	JF893901
A. phagocytophilum	S1071/08	JF893900
A. phagocytophilum	S654/04	JF893899
A. phagocytophilum	Cc20	FJ812387
A. phagocytophilum	Ce9	FJ812384
A. phagocytophilum	NY18	AY164513
A. phagocytophilum	6497	JN656334
A. phagocytophilum	9774	JN656332
A. phagocytophilum	630300	JN656333
A. phagocytophilum	8776	JN656331
A. phagocytophilum	232	JN656330
A. phagocytophilum	15526	JN656329
A. phagocytophilum	6385	JN656328
A. phagocytophilum	S2872/07	JN656327

A. phagocytophilum	2838	JN656326
A. phagocytophilum	S1710/05	JN656325
A. phagocytophilum	8340	JN656324
A. phagocytophilum	14524	JN656323
A. phagocytophilum	50	JN656319
A. phagocytophilum	6380	JN656318
A. phagocytophilum	633200	JN656317
A. phagocytophilum	KD	JN656316
A. phagocytophilum	4	JN656315
A. phagocytophilum	67	JN656314
A. phagocytophilum	1219	JN656313
A. phagocytophilum	614	JN656312
A. phagocytophilum	14908	JN656310
A. phagocytophilum	20684	JN656311
A. phagocytophilum	3547	JN656309
A. phagocytophilum	754	JN656308
A. phagocytophilum	7444	JN656307
A. phagocytophilum	8074	JN656306
A. phagocytophilum	29W	JN656304
A. phagocytophilum	20489	JN656305
A. phagocytophilum	S2070/03	JN656303
A. phagocytophilum	152595	JN656302
A. phagocytophilum	680200	JN656301
A. phagocytophilum	44	JN656300
A. phagocytophilum	19555	JN656299
A. phagocytophilum	9698	JN656298
A. phagocytophilum	14888	JN656297
A. phagocytophilum	R1592	JN244019
A. phagocytophilum	ovine 5	AY706393
A. phagocytophilum	Elsa	AY706392
A. phagocytophilum	JM	CP006617
A. phagocytophilum	HZ2	CP006616

Supplementary Table S10. GenBank® accession codes for the *rrhyp* gene sequences of *Rickettsia ricketsii*. Sequences utilized for *in silico* analysis in Supplementary Fig. S1 are emphasized in bold.

***Rickettsia* Species**	**Isolate/Strain**	**GeneBank®**
R. rickettsii	Iowa	CP018914
R. rickettsii	Iowa	CP018913
R. rickettsii	Morgan	CP006010
R. rickettsii	Iowa	CP000766
R. rickettsii	Hauke	CP003318
R. rickettsii	Hino	CP003309
R. rickettsii	Arizona	CP003307
R. rickettsii	Colombia	CP003306
R. rickettsii	Brazil	CP003305
R. rickettsii	R	CP006009
R. rickettsii	**Sheila Smith**	**CP000848**
R. rickettsii	Hlp#2	CP003311

Supplementary Table S11. Paired t-test statistical analysis of *Borrelia* species with singleplex (SP) and Layerplex (LP) qPCR testing. Mean values reported as quantification cycles (Cq) from triplicate testing.

	B. hermsii		*B. turicatae*		*B. parkeri*		*B. burgdorferi*	
	SP	LP	SP	LP	SP	LP	SP	LP
Mean	26.040	27.306	23.550	25.713	26.018	25.912	25.061	25.297
Variance	1.133	0.254	0.245	0.191	0.014	0.014	0.003	0.014
Observations	3	3	3	3	3	3	3	3
P(T≤) two-tail	0.068		0.723		0.423		0.125	

Supplementary Table S12. Paired t-test statistical analysis of *Ehrlichia, Anaplasma, and Rickettsia* species with singleplex (SP) and layerplex (LP) qPCR testing. Mean values reported as quantification cycles (Cq) from triplicate testing.

	E. canis		*E. chaffeensis*		*E. ewingii*		*A. phagocytophilum*		*R. rickettsii*	
	SP	LP	SP	LP	SP	LP	SP	LP	SP	LP
Mean	25.434	25.253	25.429	25.475	25.387	25.280	25.906	25.894	25.302	25.394
Variance	0.017	0.032	0.011	0.005	0.005	0.003	0.280	0.091	0.001	0.017
Observations	3	3	3	3	3	3	3	3	3	3
P(T≤) two-tail	0.252		0.207		0.146		0.941		0.272	

Supplementary Table S13. Paired t-test statistical analysis of *Babesia* species with singleplex (SP) and layerplex (LP) qPCR testing. Mean values reported as quantification cycles (Cq) from triplicate testing.

	Babesia spp.	
	SP	LP
Mean	25.196	25.126
Variance	0.001	0.001
Observations	3	3
P(T≤) two-tail	0.135	

Supplementary Table S14. Paired t-test statistical analysis of the canine specific endogenous internal positive control (EIPC-K9) with singleplex (SP) and layerplex (LP) qPCR testing. Mean values reported as quantification cycles (Cq) from triplicate testing.

	EIPC-K9	
	SP	LP
Mean	25.673	25.175
Variance	0.026	0.166
Observations	3	3
P(T≤) two-tail	0.093	
